# IL-20RB mediates tumoral response to osteoclastic niches and promotes bone metastasis of lung cancer

**DOI:** 10.1172/JCI157917

**Published:** 2022-10-17

**Authors:** Yunfei He, Wenqian Luo, Yingjie Liu, Yuan Wang, Chengxin Ma, Qiuyao Wu, Pu Tian, Dasa He, Zhenchang Jia, Xianzhe Lv, Yu-Shui Ma, Haitang Yang, Ke Xu, Xue Zhang, Yansen Xiao, Peiyuan Zhang, Yajun Liang, Da Fu, Feng Yao, Guohong Hu

**Affiliations:** 1 Shanghai Institute of Nutrition and Health, University of Chinese Academy of Sciences, Chinese Academy of Sciences, Shanghai, China.; 2 Department of Nuclear Medicine, Shanghai Tenth People’s Hospital, Tongji University School of Medicine, Shanghai, China.; 3 Department of Thoracic Surgery, Shanghai Chest Hospital, Shanghai Jiaotong University, Shanghai, China.; 4 General Surgery, Ruijin Hospital and Institute of Pancreatic Diseases, Shanghai Jiaotong University School of Medicine, Shanghai, China.

**Keywords:** Cell Biology, Cancer

## Abstract

Bone is a common site of metastasis in lung cancer, but the regulatory mechanism remains incompletely understood. Osteoclasts are known to play crucial roles in osteolytic bone metastasis by digesting bone matrix and indirectly enhancing tumor colonization. In this study, we found that IL receptor 20 subunit β (IL-20RB) mediated a direct tumoral response to osteoclasts. Tumoral expression of IL-20RB was associated with bone metastasis of lung cancer, and functionally, IL-20RB promoted metastatic growth of lung cancer cells in bone. Mechanistically, tumor cells induced osteoclasts to secrete the IL-20RB ligand IL-19, and IL-19 stimulated IL-20RB–expressing tumor cells to activate downstream JAK1/STAT3 signaling, leading to enhanced proliferation of tumor cells in bone. Importantly, blocking IL-20RB with a neutralizing antibody significantly suppressed bone metastasis of lung cancer. Overall, our data revealed a direct protumor role of osteoclastic niche in bone metastasis and supported IL-20RB–targeting approaches for metastasis treatment.

## Graphical abstract

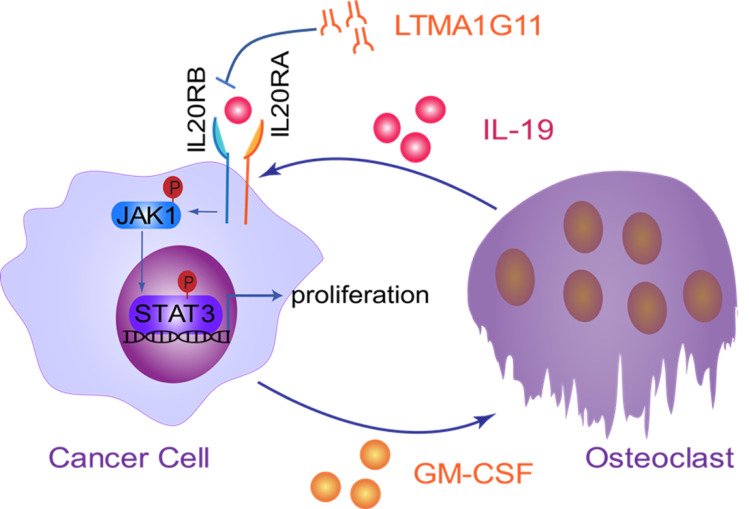

## Introduction

Lung cancer is the leading cause of cancer-related deaths (1) and often recurs in distant organs. Skeleton is the most frequent target site of distant metastasis for lung cancer. Up to 40% patients with non–small cell lung cancer (NSCLC) develop bone metastases, with a reported average survival time of 6 to 10 months after metastasis (2, 3). Even worse, 5-year survival for these patients with current therapies is less than 5% (4). In addition to higher mortality, bone metastasis also causes physical torment, including pain and fractures, and greatly reduces the quality of life of patients (5–7). Thus, further understanding of the mechanisms underlying lung-to-bone metastasis to support designing of new metastasis-targeting remedies is of clinical significance.

The current comprehension of osteolytic bone metastasis of various cancer types, including lung cancer and breast cancer, underscores the central roles of osteoclasts in the osseous environment (8). Tumor cells induce the differentiation of osteoclasts from mononuclear precursors by producing osteoclast-stimulating factors or by engaging other stromal cells, such as osteoblasts (9–17); in turn, osteoclasts digest bone matrix, leading to the release of embedded growth factors, such as TGF-β, IGF, and calcium, to facilitate tumor cell seeding and proliferation (18–24). The functional importance of osteoclasts is further supported by the development and clinical application of osteoclast inhibitors, including bisphosphonates (25, 26) and denosumab (27, 28), to treat bone metastasis. However, the involvement of osteoclasts in bone metastasis is far from being completely understood. Other than contributing to bone resorption, osteoclasts might also act on other stromal or tumoral components in bone. For example, recently it has been found that osteoclasts inhibit T cell–mediated cytotoxicity and thus indirectly promote the survival of multiple myeloma cells (29). In addition, osteoclasts could also secrete protumor lipid factors to regulate breast cancer bone metastasis (30). These studies indicate a versatile but underexplored role of osteoclasts.

IL receptor 20 subunit β (IL-20RB) forms a heterodimer with the α subunit IL-20RA or IL receptor 22 (IL-22R) to bind to IL-20 subfamily cytokines, including IL-19, IL-20, and IL-24 (31, 32). Upon ligand binding, the heterodimeric receptor activates the intracellular JAK1/STAT3 signaling pathway (32–34). The IL-20 cytokines play essential roles in innate immunity and tissue hemostasis and are found to be upregulated in various human diseases, such as psoriasis and rheumatoid arthritis (35–37). Recent studies have also shown the regulation of IL-20 signaling in cancer development. IL-20 has been reported to promote the progression and metastasis of prostate cancer, oral cancer, and breast cancer (38–40). In addition, IL-19 is highly expressed in breast cancer and is associated with a poor clinical outcome (41). Aberrant expression of IL-20RB has also been reported in various cancer types (42–44). However, the involvement of IL-20RB in bone metastasis is unknown.

In this study, we show that the IL-19/IL-20RB axis mediates a direct osteoclastic stimulus to tumor to enhance the proliferation of disseminated lung cancer cells in bone and that IL-20RB can be targeted to treat lung cancer bone metastasis.

## Results

### IL-20RB expression is associated with bone metastasis of lung cancer.

**Figure 1:**
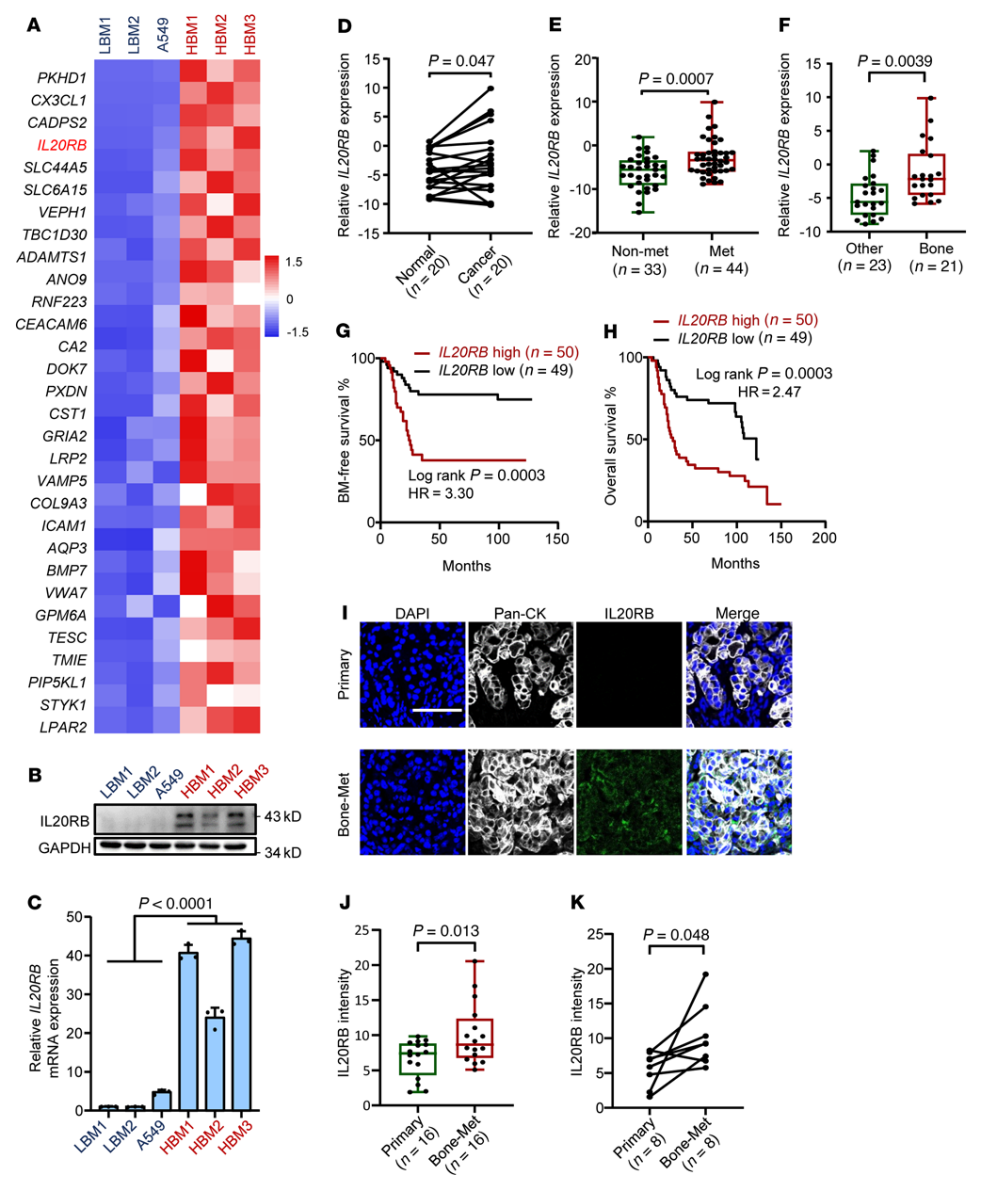
*IL20RB* expression is associated with lung cancer bone metastasis. (**A**) Expression heatmap of upregulated genes in highly (red) versus weakly/mildly (blue) bone-metastatic A549-derivative cell lines. (**B** and **C**) IL-20RB protein (**B**) and mRNA levels (**C**) in A549-derivative cell lines. (**D**–**K**) Analysis of *IL20RB* expression in an SHTCH lung cancer cohort. *IL20RB* expression in paired normal and cancer tissues from the same patients (**D**), in metastatic and nonmetastatic primary lung tumors (**E**), and in primary lung tumors metastatic to bone or other organs (**F**). Bone metastasis–free survival (**G**) and overall survival (**H**) analyses of the patients according to different *IL20RB* expression status. Representative images (**I**) and quantitation (**J**) of IL-20RB immunostaining in primary lung tumors and bone metastases and in paired lung tumors and bone metastases from the same patients (**K**). Tumor cells were stained by pan-cytokeratin (CK). Scale bar: 100 μm. *P* values were obtained by 2-tailed paired (**D** and **K**) or unpaired *t* test (**C**, **E**, **F**, and **J**) and log-rank test (**G** and **H**). Box plots display values of minimum, first quartile, median, third quartile, and maximum. Data are represented as mean ± SD.

To study lung cancer bone metastasis, we established a series of A549-derivative cell lines with varied metastatic abilities to bone. By in vivo selection in mice and single cell progeny cloning from the mildly metastatic parental A549 cells (Supplemental Figure 1A; supplemental material available online with this article; https://doi.org/10.1172/JCI157917DS1), 3 highly bone-metastatic derivatives (HBM1, HBM2, and HBM3) and 2 weakly bone-metastatic derivatives (LBM1 and LBM2) were generated (Supplemental Figure 1, B and C). The metastatic cell lines caused manifested osteolysis and metastasis following intracardiac inoculation into mice (Supplemental Figure 1, B and C). We next carried out an RNA-Seq analysis of the parental and derivative lines and paid particular attention to the gene-encoding secretory and cell-membrane proteins (Figure 1A), which often play critical roles in tumor-microenvironment interaction. Then we assessed the clinical relevance of the differentially expressed genes by analyzing a previously published Korean lung cancer clinical data set (45) and the UCSC Xena lung cancer data set (46). *IL20RB*, but not several other top candidate genes, such as *PKHD1*, *CX3CL1*, or *CADPS2,* was markedly upregulated in lung tumors as compared with normal lung tissues and linked to shortened patient survival time (Supplemental Figure 1, D–H). In addition, the expression of *IL20RA*, the gene encoding the α subunit of IL-20R, was neither obviously regulated in lung cancer nor correlated to patient survival (Supplemental Figure 1, I– J). The upregulation of IL-20RB in bone-tropic A549 sublines was confirmed in RNA and protein levels (Figure 1, B and C).

Then, the clinical relevance of *IL20RB* was further validated in a Shanghai Tenth People’s Hospital and Shanghai Chest Hospital (SHTCH) lung cancer cohort. The analysis revealed a significant elevation of *IL20RB* expression in tumor tissues compared with that in the paratumor normal tissues of the same patients (Figure 1D). *IL20RB* was also upregulated in metastatic tumors more than in nonmetastatic tumors (Figure 1E). Among the metastatic tumors, bone-tropic tumors expressed *IL20RB* at much higher levels than the tumors prone to metastasis to other organs (Figure 1F). In addition, patients with higher *IL20RB* expression had a higher risk of bone recurrence (Figure 1G) and poorer overall survival (Figure 1H). Furthermore, immunostaining showed that the expression of IL-20RB in bone metastases of lung cancer was significantly higher than in primary lung tumors (Figure 1, I and J), which was also observed in paired bone metastases and primary tumors of the same patients (Figure 1K). These findings reveal a link between *IL20RB* and bone metastasis in lung cancer.

We next asked how *IL20RB* was upregulated in bone-tropic cells. We analyzed the promoter region of IL-20RB by JASPAR (47) and found multiple binding sites of FOXP2, a forkhead family transcription factor, on the promoter (Supplemental Figure 2, A and B). In addition, FOXP2 was also upregulated in the bone-tropic A549 sublines (Supplemental Figure 2C). Importantly, *FOXP2* knockdown by shRNAs led to suppression of *IL20RB* in HBM1 (Supplemental Figure 2D), indicating that *IL20RB* might be regulated by *FOXP2* in lung cancer.

### IL-20RB enhances proliferation of tumor cells in bone and promotes bone metastasis.

**Figure 2:**
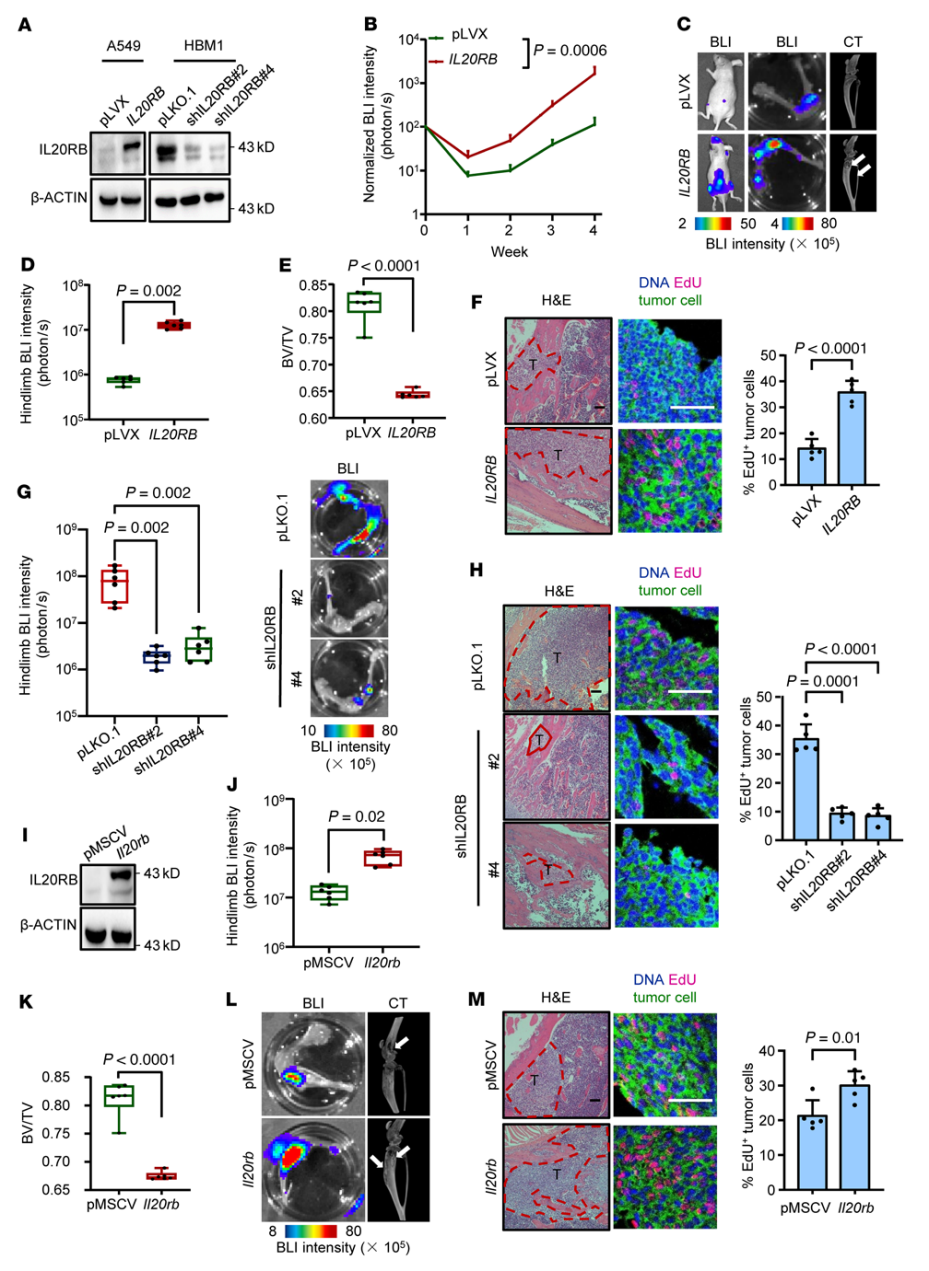
IL-20RB promotes bone metastasis of lung cancer. (**A**) *IL20RB* overexpression in A549 and knockdown in HBM1. (**B**–**F**) Intracardiac injection of A549 with or without *IL20RB* overexpression into nude mice for bone metastasis analysis. Weekly BLI quantitation of tumor burden of the mice (**B**, *n* = 7 mice per group), representative images of BLI analyses of whole bodies and hind limbs and micro-CT analyses for bone destruction of hind limbs (**C**, arrows point to osteolytic areas), ex vivo BLI analysis of hind limbs (**D**), micro-CT quantification of relative bone volumes of hind limbs (**E**), and H&E and EdU labeling analysis of bone metastases (**F**). (**G** and **H**) Intracardiac injection of HBM1 cells with *IL20RB* knockdown for bone metastasis analysis (*n* = 6, pLKO.1; *n* = 7, shIL20RB#2; *n* = 7, shIL20RB#4). Ex vivo hind limb BLI analysis (**G**) and H&E- and EdU-labeling analysis of bone metastases (**H**). (**I**) *Il20rb* overexpression in LLC. (**J**–**M**) Intracardiac injection of LLC with or without *Il20rb* overexpression for bone metastasis analysis (*n* = 6 mice per group). Ex vivo BLI quantification of hind limbs (**J**), micro-CT quantification of relative bone volumes of hind limbs (**K**), representative BLI and micro-CT images of hind limbs (**L**, arrows point to osteolytic areas), and H&E- and EdU–labeling analysis of bone metastases (**M**). BV/TV, bone volume/total volume; T, tumor; B, bone. Scale bars: 100 μm. *P* values were obtained by Mann-Whitney *U* test (**B**, **D**, **G**, and **J**) and 2-tailed unpaired *t* test (**E**, **F**, **H**, **K**, and **M**). Box plots display values of minimum, first quartile, median, third quartile, and maximum. Data are represented as mean ± SD.

To explore the functional role of IL-20RB in bone metastasis, *IL20RB* was stably overexpressed in A549 and knocked down in the bone-tropic HBM1 (Figure 2A). When these cancer cells were intracardiacally inoculated into immunodeficient nude mice, *IL20RB* overexpression significantly accelerated bone metastasis, resulting in exacerbated bone damage (Figure 2, B–F), but had no influence on the metastasis to other nonbone organs (Supplemental Figure 3, A and B). In addition, *IL20RB* knockdown reduced bone-metastatic burden and bone resorption by HBM1 cells (Supplemental Figure 3, C–E, and Figure 2, G and H). *IL20RB* knockdown in another NSCLC cell line, H460, which was known to be bone metastatic (48) and expressed a relatively high level of *IL20RB* (Supplemental Figure 3F), also led to obvious suppression of bone metastasis in vivo (Supplemental Figure 3, G–J). We further tested the effect of IL-20RB on bone metastasis in immunocompetent mice by overexpressing murine *Il20rb* in Lewis lung cancer (LLC) cells (Figure 2I). Concordantly, the bone metastasis burden caused by *Il20rb*-overexpressing LLC cells in C57 mice was found to be markedly heavier than that caused by control cells (Figure 2, J–M). These findings revealed a probone metastasis role of *IL20RB* in lung cancer.

Along with the role of IL-20RB in bone metastasis, we found that IL-20RB expression led to higher proliferative capacity of lung tumor cells in bone. By 5-ethynyl-2′-deoxyuridine (EdU) labeling 24 hours prior to bone harvest from the mice, it was found that higher percentages of *IL20RB*-overexpressing tumor cells were active in proliferation, while knockdown of *IL20RB* suppressed proliferation of tumor cells in bone (Figure 2, F, H, and M). Together, these data demonstrate that IL-20RB promotes the proliferation of lung tumor cells in bone and enhances metastatic colonization.

### Osteoclast-secreted IL-19 primes proliferation of IL-20RB–expressing tumor cells.

**Figure 3:**
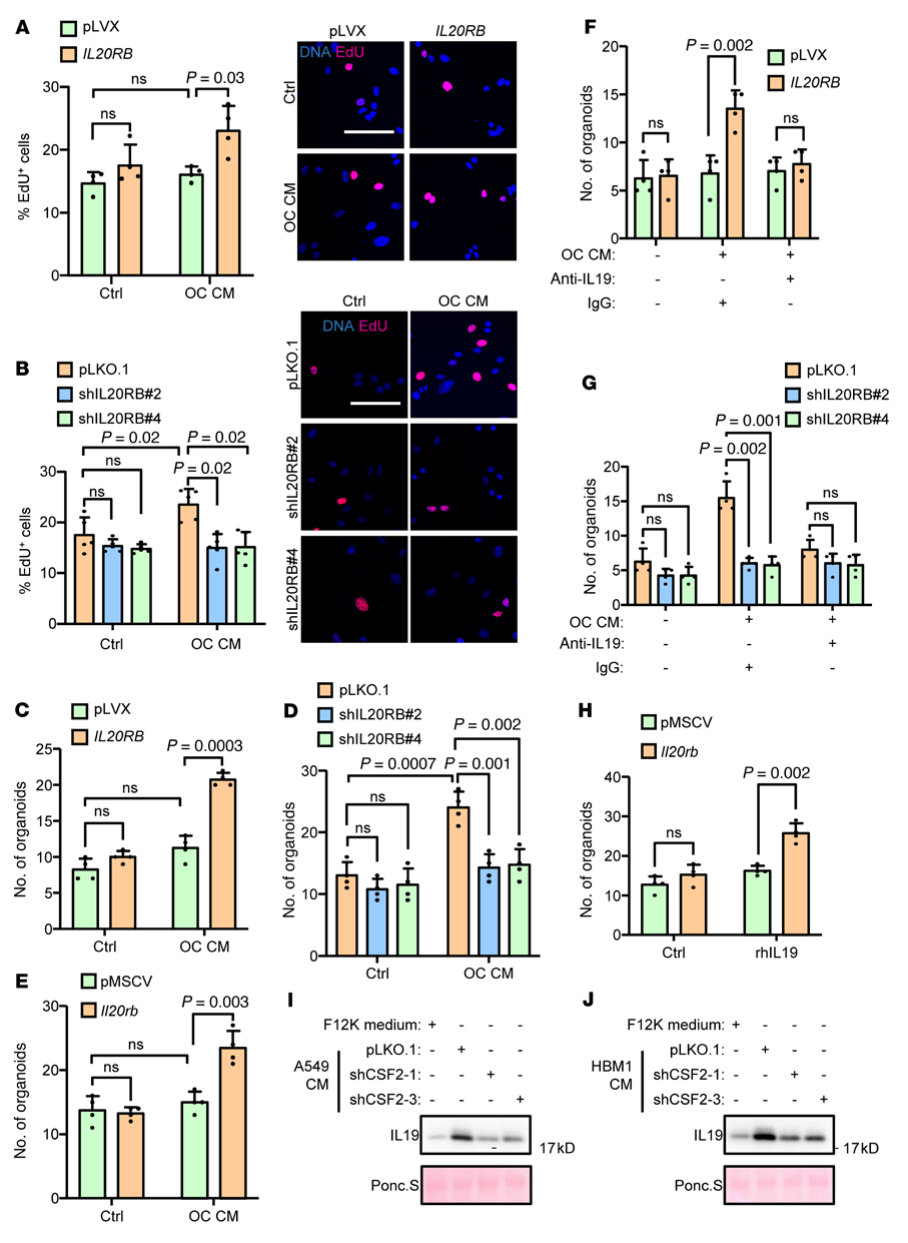
Osteoclast-secreted IL-19 primes *IL20RB*-expressing tumor cells for proliferation in bone. (**A**) Quantification of EdU^+^ A549 cells with *IL20RB* overexpression and/or treatment of CM from murine bone marrow–derived osteoclasts (OC) for 24 hours. Representative images are shown on the right. OC CM was mixed with A549 culture medium at a 1:3 ratio. (**B**) Quantification of EdU^+^ HBM1 cells with *IL20RB* knockdown and/or treatment with OC CM for 24 hours. (**C**–**E**) Organoid formation of A549 (**C**), HBM1 (**D**), and LLC (**E**) after treatment with OC CM. OC CM was mixed with organoid culture medium at a 1:3 ratio. (**F** and **G**) Organoid formation of A549 (**F**) and HBM1 (**G**) after treatment with OC CM and/or the IL-19–neutralizing antibody (10 μg/mL). (**H**) Organoid formation of LLC cells after treatment with recombinant IL-19 protein. (**I** and **J**) IL-19 secretion of osteoclasts after treatment with control F12K medium or CM from A549 (**I**) or HBM1 (**J**) with or without *CSF2* knockdown. Scale bars: 100 μm. *P* values were obtained by 2-tailed unpaired *t* test. Data are represented as mean ± SD.

Thus, we investigated how IL-20RB regulated bone metastasis. As tumor cells often induce osteoclastogenesis for bone colonization, we analyzed the presence of mature osteoclasts in bone lesions by tartrate-resistant acid phosphatase (TRAP) staining. Surprisingly, neither *IL20RB* knockdown nor overexpression in tumor cells obviously altered the abundance of TRAP^+^ osteoclasts along the tumor-bone interface (Supplemental Figure 4, A–C). Concordantly, in vitro osteoclastogenesis assays also revealed that IL-20RB did not influence osteoclastic differentiation from murine primary bone marrow (Supplemental Figure 4, D–F). However, when A549 cells were treated with conditioned medium (CM) of osteoclasts, the proliferation of *IL20RB*-overexpressing tumor cells, as measured by EdU labeling, was markedly stimulated by the CM, while the control A549 cells demonstrated no response from osteoclastic CM (Figure 3A). Reciprocally, the metastatic HBM1 cells were responsive to the osteoclastic stimulus, but the response was reduced after *IL20RB* was knocked down (Figure 3B). The responsiveness of tumor cells in proliferation to osteoclasts was further verified with 3D tumor organoid assays to recapitulate the in vivo tumor architecture and phenotypes (49–51). *IL20RB* expression significantly enhanced organoid growth of human and murine tumor cells with the treatment of osteoclastic CM (Figure 3, C–E, and Supplemental Figure 5A). These data indicated that *IL20RB* mediates a proproliferative response of tumor cells toward osteoclasts rather than regulating tumoral induction of osteoclastogenesis. To rule out the possibility of an autocrine effect of IL-20RB on tumor proliferation, we compared the proliferation of the A549 sublines with different IL-20RB expression levels and found no difference in growth rates (Supplemental Figure 5B). In addition, cancer cells incubated in the CM of control or *IL20RB*-overexpressing A549 cells demonstrated no proliferative difference (Supplemental Figure 5C), indicating that IL-20RB mediated the tumoral response to a paracrine factor.

Then we sought to find out which factor in osteoclastic CM triggers the proliferative response of cancer cells. The ligands of IL-20R include IL-19, IL-20, and IL-24. We found that only IL-19 was abundantly expressed by osteoclasts, while the other 2 ligands were barely expressed (Supplemental Figure 5, D and E). In addition, tumor cells expressed a much lower level of IL-19 than osteoclasts (Supplemental Figure 5, D and E). Importantly, immunostaining of clinical bone metastases of lung cancer showed abundant expression of IL-20RB in tumor cells and IL-19 in microenvironmental cells (Supplemental Figure 5F). These data indicated that osteoclast-derived IL-19 might be the key factor in stimulating lung cancer cells. Confirming this hypothesis, administration of an IL-19–neutralizing antibody in the osteoclastic CM blocked the effect of CM in promoting organoid formation of *IL20RB*-expressing tumor cells (Figure 3, F and G). In addition, the recombinant IL-19 protein was able to enhance organoid formation by A549, HBM1, H460, and LLC cells when these cells expressed *IL20RB*. *IL20RB* knockdown impaired the responsiveness of tumor cells to recombinant IL-19 (Figure 3H and Supplemental Figure 5, G–J).

Interestingly, when osteoclasts were incubated in tumor cell CM, IL-19 secretion by osteoclasts was enhanced by CM of both A549 and HBM1 (Figure 3, I and J). As it has been reported that GM-CSF can induce the expression of IL-19 (52) and tumor cells often produce GM-CSF abundantly, we tested to determine whether osteoclastic IL-19 expression was regulated by GM-CSF. Indeed, A549, HBM1, and LLC cells all secreted GM-CSF (Supplemental Figure 5, K and L). In addition, when the GM-CSF gene *CSF2* was knocked down in A549 and HBM1 to suppress GM-CSF secretion (Supplemental Figure 5K), the effect of their CM to promote osteoclastic IL-19 production was diminished (Figure 3, I and J). *CSF2* knockdown also suppressed tumor-induced osteoclast maturation (Supplemental Figure 5M), corroborating a role of GM-CSF in osteoclastogenesis. Taken together, these data suggest that tumor cells can induce the secretion of IL-19 from osteoclasts and that osteoclast-derived IL-19 in turn facilitates the proliferation of *IL20RB*-expressing lung cancer cells.

### Il19 KO in mice suppresses lung cancer bone metastasis.

**Figure 4:**
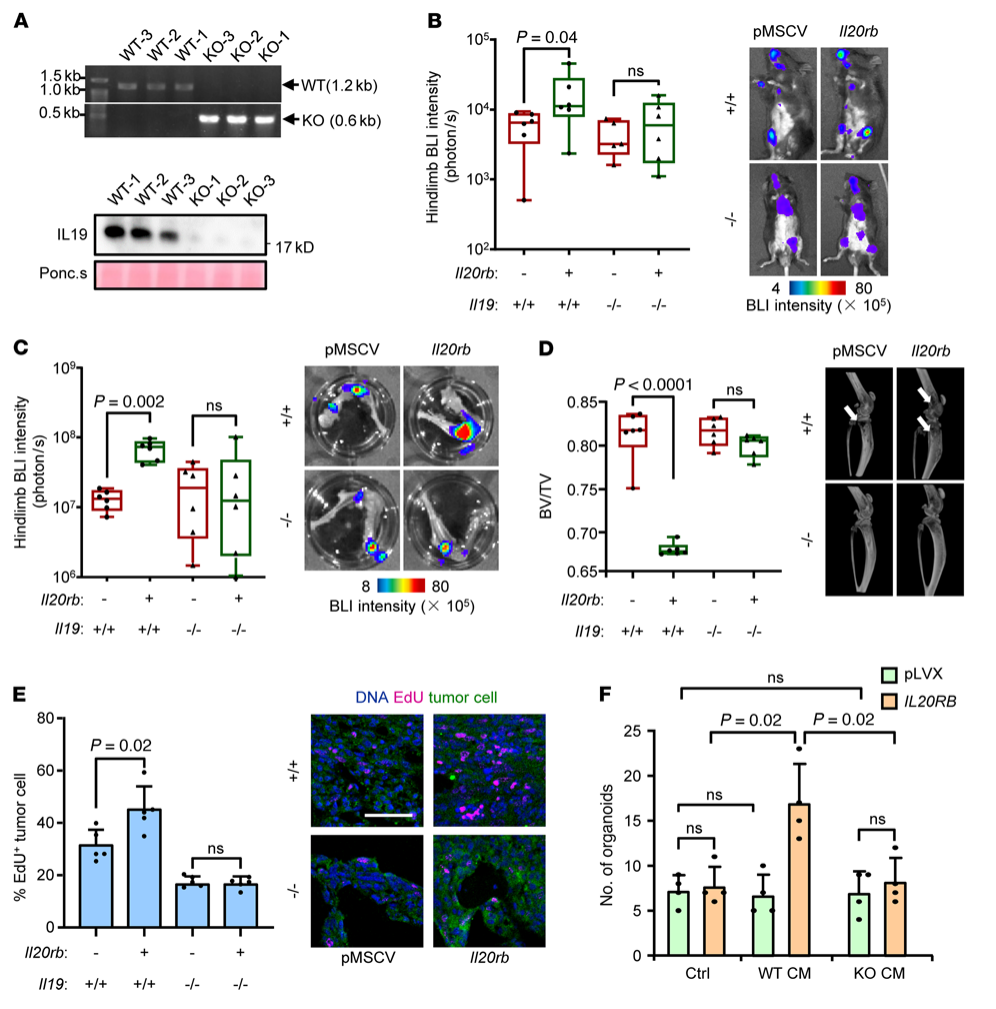
*Il19* knockout in host mice suppresses lung cancer bone metastasis. (**A**) Genotyping (top) and IL-19 secretion by bone marrow cells (bottom) of *Il19* WT (+/+) or KO (–/–) mice. (**B**–**E**) Intracardiac injection of LLC with or without *Il20rb* overexpression into *Il19* WT or KO mice for bone metastasis analysis. Whole-body BLI analysis of tumor burden (**B**), ex vivo BLI analysis of hind limbs (**C**), micro-CT analysis of hind limbs (**D**, arrows point to osteolytic areas), and immunofluorescent (IF) analysis of EdU^+^ tumor cells in bone (**E**). (**F**) Organoid formation of A549 cells (with or without *IL20RB* overexpression) after treatment with OC CM from WT or KO mice. Scale bars: 100 μm. *P* values were obtained by Mann-Whitney *U* test (**B** and **C**) and 2-tailed unpaired *t* test (**D**–**F**). Scale bar: 100 μm. Data are represented as mean ± SD. Box plots display values of minimum, first quartile, median, third quartile, and maximum.

In order to further verify the role of the IL-19/IL-20RB axis in bone metastasis, an *Il19*-KO mouse strain was generated by deleting the region of exons 2 to 5 of *Il19* via a CRISPR-Cas9 system (Supplemental Figure 6A and Figure 4A). A decrease in *Il19* expression in bone marrow and a variety of other tissues of KO mice was confirmed by Western blotting and quantitative PCR (qPCR) (Figure 4A and Supplemental Figure 6B). The KO mice were viable and fertile and exhibited no apparent abnormalities compared with their WT littermates (Supplemental Figure 6C). We then inoculated *Il20rb*-overexpressing or control LLC cells into the left cardiac ventricle of KO mice or WT mice. *Il20rb* overexpression enhanced bone metastasis in WT mice. However, *Il19* KO in host mice led to suppression of bone metastasis (Figure 4, B–D). Importantly, *Il20rb* overexpression in tumor cells had no effect on bone metastasis in *Il19*-KO mice (Figure 4, B–D). IL-20RB–promoted tumor proliferation in bone metastases was also diminished by *Il19* KO of host mice (Figure 4E). In addition, when primary bone marrow cells were isolated from WT and KO mice, they showed no obvious difference in differentiation into mature osteoclasts (Supplemental Figure 6D). However, the CM of *Il19*-KO osteoclasts lost the ability to facilitate organoid formation of *IL20RB*-overexpressing A549 cells (Figure 4F). These data indicate that both microenvironment-derived IL-19 and tumor expression of IL-20RB are crucial for bone metastasis of lung cancer.

### Osteoclast-secreted IL-19 activates JAK1/STAT3 signaling in IL20RB-expressing tumor cells.

**Figure 5:**
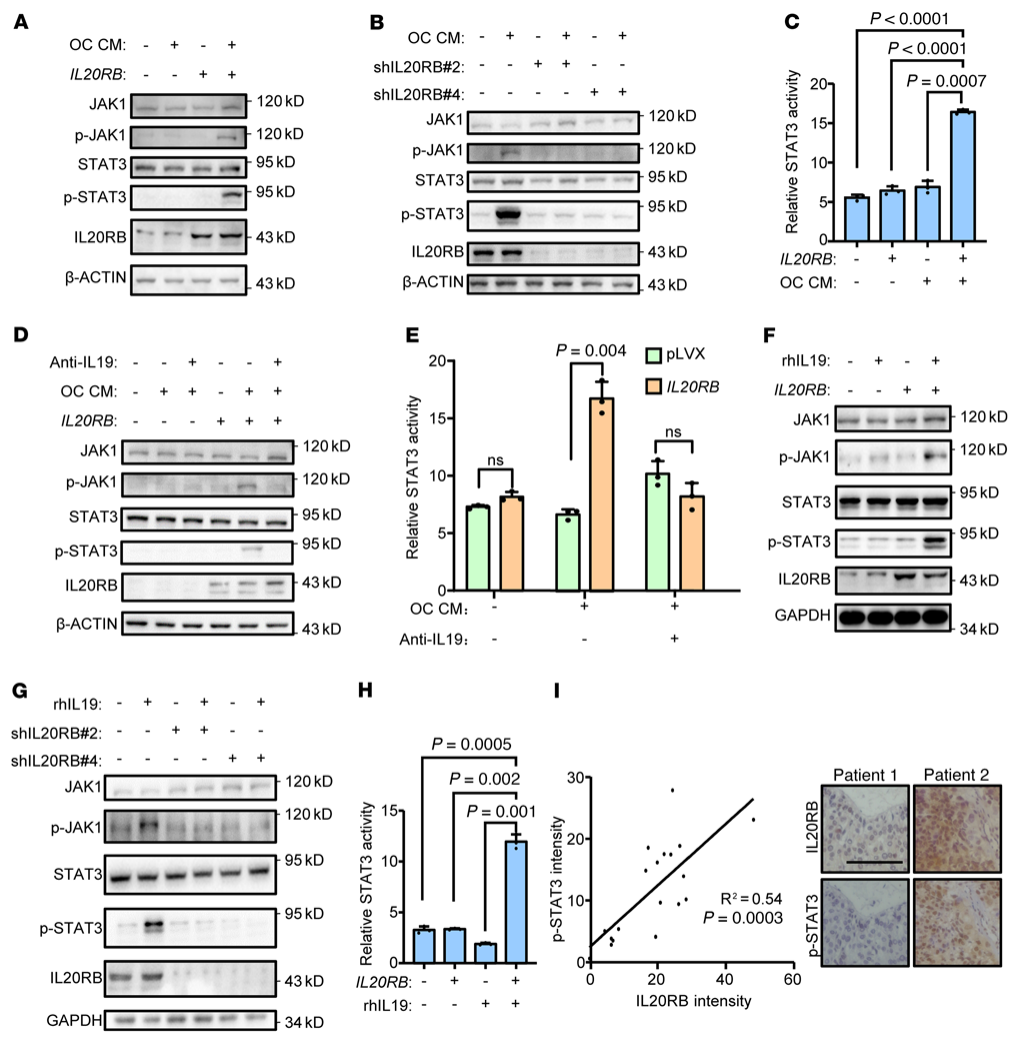
Osteoclast-secreted IL-19 activates JAK1/STAT3 signaling in *IL20RB*-expressing tumor cells. (**A** and **B**) Phosphorylation of JAK1 and STAT3 in A549 with or without *IL20RB* overexpression (**A**) and HBM1 with or without *IL20RB* knockdown (**B**) after treatment with OC CM for 24 hours. OC CM was mixed with A549 culture medium at a 1:3 ratio. (**C**) STAT3-responsive reporter activity in A549 with or without *IL20RB* overexpression after treatment with OC CM for 24 hours. (**D** and **E**) Phosphorylation of JAK1 and STAT3 (**D**) and STAT3-responsive reporter activity (**E**) in A549 with or without *IL20RB* overexpression after treatment with OC CM and/or IL-19–neutralizing antibody (10 μg/mL) for 24 hours. (**F** and **G**) Phosphorylation of JAK1 and STAT3 in A549 with or without *IL20RB* overexpression (**F**) and HBM1 with or without *IL20RB* knockdown (**G**) after treatment with IL-19 recombinant protein (25 ng/mL) for 15 minutes. (**H**) STAT3-responsive reporter activity in A549 with or without *IL20RB* overexpression after treatment with IL-19 recombinant protein (25 ng/mL) for 15 minutes. (**I**) Correlation between IL-20RB expression and STAT3 phosphorylation in the SHTCH lung cancer cohort (*n* = 19 patients). Representative immunostaining images are shown on the right. Scale bar: 100 μm. *P* values were obtained by 2-tailed unpaired *t* test (**C**, **E**, and **H**) and Pearson’s correlation analysis (**I**). Data are represented as mean ± SD.

IL-20 subfamily cytokines have been shown to activate the JAK1/STAT3 signaling pathway (32–34), which plays a crucial role in cancer cell proliferation and tumor progression (53, 54). Thus, we tested to determine whether osteoclast-derived IL-19 activates STAT3 in lung cancer cells. Incubating tumor cells in osteoclastic CM led to a noticeable rise of JAK1 and STAT3 phosphorylation in *IL20RB*-overexpressing A549 cells, but not in control A549 cells (Figure 5A). In contrast, osteoclast-induced JAK1 and STAT3 phosphorylation in HBM1 cells apparently declined when *IL20RB* was knocked down (Figure 5B). The similar phenomenon was also observed in LLC and H460 cells (Supplemental Figure 7, A and B). STAT3 activation by osteoclastic CM in *IL20RB*-expressing cancer cells was also confirmed with a STAT3 luciferase reporter (55) (Figure 5C). The IL-19–neutralizing antibody effectively blocked osteoclast-induced STAT3 activation in *IL20RB*-expressing tumor cells (Figure 5, D and E). In addition, recombinant IL-19 was able to promote JAK1/STAT3 activation in A549, HBM1, H460, and LLC cells, but only when the cells expressed IL-20RB (Figure 5, F–H, and Supplemental Figure 7, C and D). Furthermore, immunostaining of clinical samples from the SHTCH lung cancer cohort also revealed a strong positive correlation between IL-20RB expression and STAT3 phosphorylation (Figure 5I). Together, these data demonstrate that JAK1/STAT3 signaling is activated in *IL20RB*-expressing lung cancer cells by osteoclast-secreted IL-19.

### STAT3 blockade suppresses IL-20RB–induced bone metastasis.

**Figure 6:**
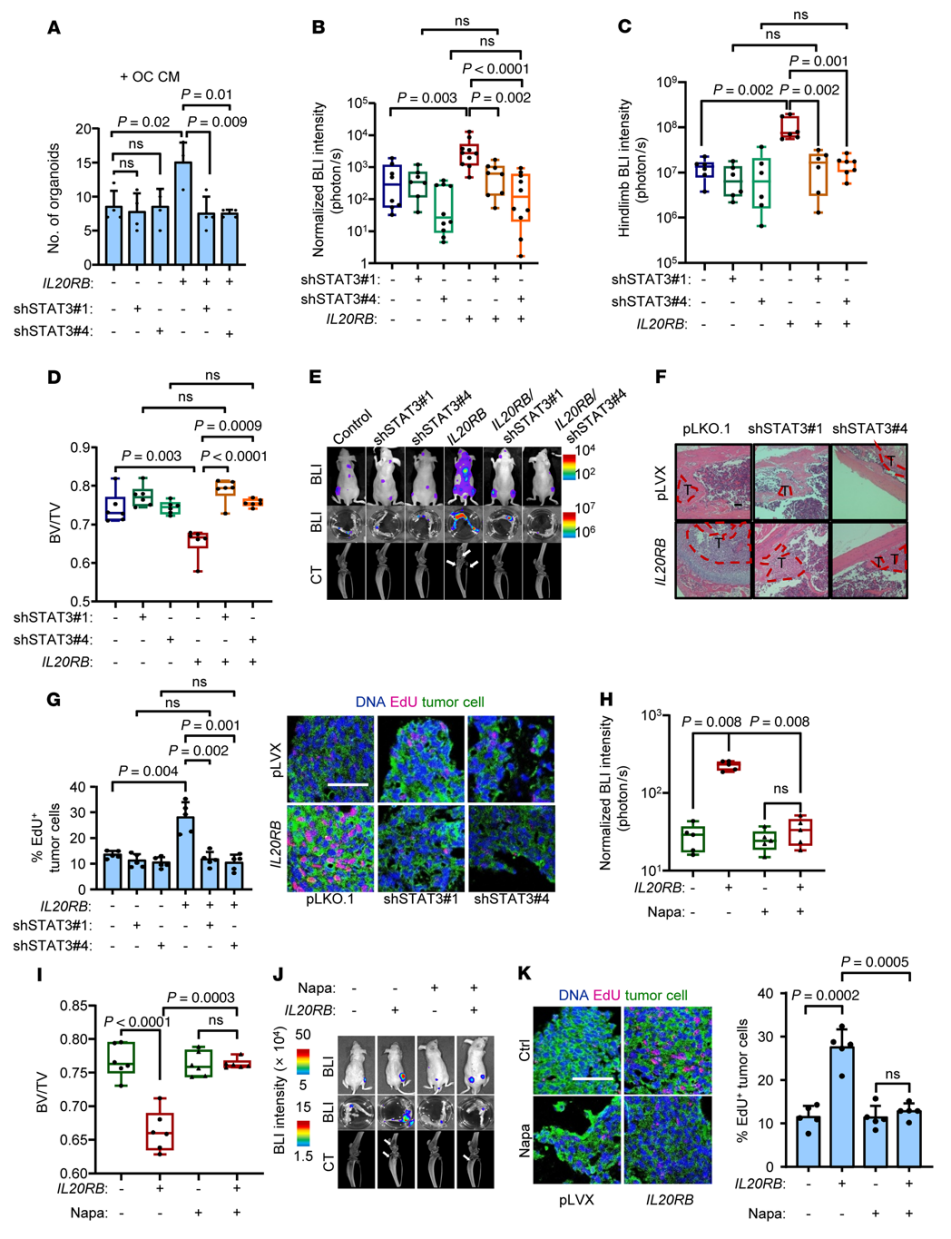
*STAT3* inhibition suppresses IL-20RB–induced tumor proliferation and bone metastasis. (**A**) Organoid formation of A549 with *IL20RB* overexpression and/or *STAT3* knockdown after treatment of OC CM. (**B**–**G**) Intracardiac injection of A549 with *IL20RB* overexpression and/or *STAT3* knockdown into nude mice for bone metastasis analysis (*n* ≥ 7 mice per group). Whole-body BLI quantification at week 4 after tumor inoculation (**B**), ex vivo hind limb BLI quantification (**C**), micro-CT quantification of relative bone volumes of hind limbs (**D**), representative BLI images of whole bodies and hind limbs, and micro-CT analyses of hind limbs (**E**, arrows point to osteolytic areas), representative H&E images of bone sections (**F**), and immunofluorescent analysis of EdU^+^ tumor cells in bone (**G**). (**H**–**K**) Napabucasin treatment of mice with intracardiac injection of control and *IL20RB*-overexpressing A549 cells for bone metastasis (*n* = 5 mice per group). Whole-body BLI quantification at week 4 after tumor inoculation (**H**), micro-CT quantification of relative bone volumes of hind limbs (**I**), representative BLI images of whole bodies and hind limbs and micro-CT analyses of hind limbs (**J**, arrows point to osteolytic areas), and immunofluorescent analysis of EdU^+^ tumor cells in bone (**K**). Scale bars: 100 μm. *P* values were obtained by Mann-Whitney *U* test (**B**, **C**, and **H**) and 2-tailed unpaired *t* test (**A**, **D**, **G**, **I**, and **K**). Box plots display values of minimum, first quartile, median, third quartile, and maximum. Data are represented as mean ± SD.

To further determine whether STAT3 acts downstream of IL-20RB in regulation of bone metastasis, *STAT3* was knocked down in both control and *IL20RB*-overexpressing A549 cells. *STAT3* knockdown abolished osteoclast- or IL-19–induced STAT3 phosphorylation in tumor cells, but did not affect upstream JAK1 phosphorylation (Supplemental Figure 8, A and B). Neither osteoclastic CM nor recombinant IL-19 protein could facilitate organoid formation of *IL20RB*-overexpressing A549 cells when *STAT3* was suppressed (Figure 6A and Supplemental Figure 8C). When A549 cells with *IL20RB* overexpression and *STAT3* knockdown were inoculated into mice, *STAT3* knockdown dramatically attenuated IL-20RB–enhanced bone metastasis (Figure 6, B–F). Tumor cell proliferation in the bone was also reduced after *STAT3* knockdown, abolishing the proproliferation effect of *IL20RB* (Figure 6G). In addition, STAT3 inhibitors, including stattic (56) and napabucasin (57, 58), suppressed STAT3 phosphorylation and activation in *IL20RB*-expressing tumor cells after stimulation by osteoclastic CM or IL-19 (Supplemental Figure 8, D–G), leading to inhibition of organoid formation (Supplemental Figure 8, H and I). More importantly, intraperitoneal treatment of the mice with napabucasin after intracardiac inoculation of *IL20RB*-expressing A549 cells was able to suppress proliferation of tumor cells disseminated in the bone, with bone lysis and bone metastasis effectively prevented (Figure 6, H–K, and Supplemental Figure 8J). Collectively, these data suggest that inhibition of STAT3 suppresses *IL20RB*-induced tumor proliferation in bone and reduces the risk of bone metastasis.

### An IL20RB-neutralizing antibody effectively suppresses bone metastasis of lung cancer.

**Figure 7:**
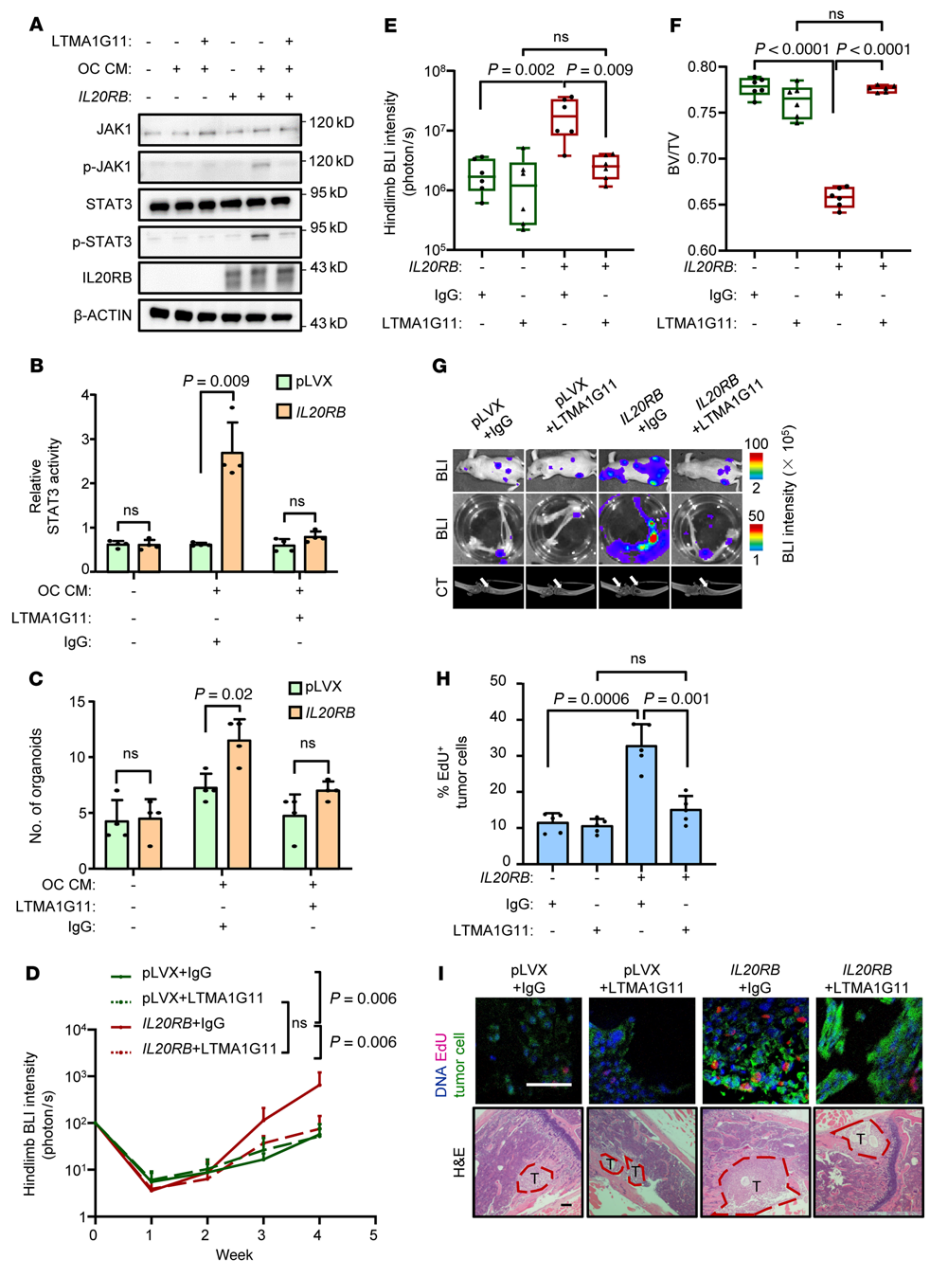
An IL-20RB–neutralizing antibody effectively suppresses bone metastasis of lung cancer. (**A**–**C**) JAK1/STAT3 phosphorylation (**A**), STAT3-responsive reporter activity (**B**), and organoid formation (**C**) of A549 with or without *IL20RB* overexpression after treatment with LTMA1G11 (5 μg/mL) and/or OC CM for 24 hours. (**D**–**I**) LTMA1G11 treatment of mice with intracardiac injection of A549 with or without *IL20RB* overexpression for bone metastasis analysis. Weekly whole-body BLI analysis (**D**, *n* = 7 mice per group), ex vivo hind limb BLI quantitation (**E**), micro-CT quantification of relative bone volumes of hind limbs (**F**), representative BLI and micro-CT images (**G**, arrows point to osteolytic areas in the legs), quantitation of EdU^+^ tumor cells (**H**) and representative H&E and immunofluorescent images of bone sections (**I**). Scale bars: 100 μm. *P* values were obtained by Mann-Whitney *U* test (**D** and **E**) and 2-tailed unpaired *t* test (**B**, **C** and **HE**). For multiple comparisons, the *P* values were corrected by the Benjamini-Hochberg procedure and were shown as *q* values (adjusted *P* values). Box plots display values of minimum, first quartile, median, third quartile, and maximum. Data are represented as mean ± SD.

The specific high expression of *IL20RB* in lung tumors and low expression in normal tissues (Figure 1D and Supplemental Figure 1D) indicate the potential of IL-20RB as a therapeutic target. Thus, we sought to develop the IL-20RB–neutralizing antibody targeting the extracellular domain of IL-20RB and successfully identified a monoclonal antibody, LTMA1G11, by functional screening. The antibody purified from LTMA1G11 hybridoma–inoculated mouse ascites (Supplemental Figure 9A) specifically detected IL-20RB in lysates of *IL20RB*-overexpressing A549 cells (Supplemental Figure 9B). Importantly, LTMA1G11 effectively inhibited the interaction between IL-19 and IL-20RB (Supplemental Figure 9C), leading to suppression of JAK1/STAT3 activation (Figure 7, A and B) and organoid formation (Figure 7C) of *IL20RB*-expressing A549 cells when the cells were treated with osteoclastic CM. Similar results were also obtained with the treatment of recombinant IL-19 protein (Supplemental Figure 9, D–F). Then we tested the efficacy of LTMA1G11 for treating bone metastasis in vivo. A549 cells with or without *IL20RB* overexpression were inoculated into the left cardiac ventricle of nude mice, followed by LTMA1G11 treatment a week later. Each animal was treated with intraperitoneal injection of 100 μg LTMA1G11 or control IgG every other day. Bioluminescent imaging (BLI) analysis showed that bone metastasis of *IL20RB*-expressing tumors was greatly reduced after treatment with LTMA1G11 (Figure 7, D–G). Three weeks after treatment, tumor burden in hind limbs was reduced by approximately 10 times, as shown by ex vivo analyses of the limbs (Figure 7, E and G). Further analyses confirmed that the treatment suppressed the proliferation of tumor cells in bone and rescued the mice from bone damage (Figure 7, F–I). We also repeated these experiments in HBM1 cells and observed similar effects of LTMA1G11. In contrast, LTMA1G11 had no effect on the metastasis of IL-20RB–deficient tumors (Supplemental Figure 9, G–J). Together, these data confirm the efficacy of the antibody for treating bone metastasis.

In addition, the drug safety of IL-20RB–targeting antibody was also tested, by treating healthy mice with LTMA1G11 in the same dosage as used in the metastasis assays, but for up to 4 weeks. It was observed that continuous LTMA1G11 treatment had no significant effect on body weights (Supplemental Figure 9K) or the blood composition of healthy mice (Supplemental Figure 9, L–N). Taken together, these results argue for the potential of IL-20RB targeting as a therapeutic strategy for treating lung cancer bone metastasis.

## Discussion

In osteolytic bone metastasis, the “vicious cycle” theory has been widely accepted (8), in which cancer cells stimulate osteoclastogenesis, and osteoclasts support cancer cell seeding and growth in bone. In this theory, osteoclasts are often regarded as “bone digester,” and the secondary effects resulting from bone resorption, including physical space for tumor growth and release of protumor factors from degraded bone matrix, facilitate tumor colonization in bone. The nonosteolytic roles of osteoclasts are less investigated. Osteoclasts can be derived from myeloid-derived suppressive cells in various pathological conditions, including cancer (59–62), indicating a possible immune-suppressive effect of osteoclasts. Indeed, recently it was reported that osteoclasts inhibit T cell immune activity in multiple myeloma (29). In addition, osteoclasts could also secrete lipids to support breast tumor cells (30). These studies indicate the versatile effects of osteoclastic niche to help tumor growth. However, it is unclear how the osteoclasts can directly interact with tumor cells for metastasis regulation in lung cancer and how the tumoral response to osteoclasts is regulated, impeding rational designing of new antiosteoclastic therapeutics.

In the present study, we reveal a critical role of the IL-19/IL-20RB/STAT3 axis to mediate the direct protumor role of osteoclastic niche. Osteoclasts secrete IL-19 ligands to act on the IL-20RB of tumor cells, thus activating the JAK1/STAT3 signaling pathway to promote proliferation of disseminated tumor cells and colonization in bone. Interestingly, tumor cells with strong or weak metastatic proclivities can all induce IL-19 secretion of osteoclasts, but their responsiveness toward osteoclasts is dependent on IL-20RB expression. This regulation of bone metastasis by responses to osteoclastic stimulus is in contrast with previously identified mechanisms in which tumor cells often demonstrate different potency of osteoclastogenesis induction (9–17). Thus, our study elucidates a mechanism of direct osteoclast-to-tumor stimulus for lung cancer bone metastasis.

In addition, the study also supports therapeutic targeting of tumoral responses to osteolytic niches, in addition to the current antiosteoclast therapeutics, for metastasis treatment. The identified IL-19/IL-20RB/STAT3 axis could be targeted at different levels. First, ligand targeting using an IL-19–neutralizing antibody markedly abolished osteoclast-induced tumor proliferation in vitro, but the in vivo efficacy, and more importantly, the possible side effects of targeting stromal-derived factors are yet to be further investigated. Targeting intracellular STAT3 with napabucasin can also remit lung cancer bone metastasis. Napabucasin is clinically approved for treating gastric cancer and in clinical trials for more malignant conditions (63, 64). Our data might indicate additional use for this drug. However, STAT3 signaling also plays vital roles in physiological processes. We noticed the deleterious effects of napabucasin treatment of the mice, including body weight loss, consistent with previous studies (65, 66). In contrast, the observation that IL-20RB is specifically expressed in lung tumors, but not in normal lung tissues, supports a receptor-targeting approach to inhibiting the tumoral response to osteoclastic stimulus. The IL-20RB–neutralizing antibody LTMA1G11 developed in this study showed promising efficacy in inhibition of bone metastasis both in vitro and in vivo. Importantly, the drug safety of the antibody was tested and no severe side effects had been found in mice. Further preclinical and clinical studies would be needed to verify the safety and efficacy of targeting IL-20RB and other components in the signaling axis to treat bone metastasis.

There are also several remaining questions regarding the IL-19/IL-20RB/STAT3 axis in bone metastasis to be answered. For example, how is IL-20RB expression regulated in lung tumor cells? Our preliminary analyses indicated a role of FOXP2, but further evidence is needed and there are possibly other upstream regulators of IL-20RB. In addition, it is still unknown whether the effect of the IL-19/IL-20RB/STAT3 axis is specific to lung cancer or also applies to other cancer types, such as breast cancer. Nevertheless, our study expands our understanding toward the roles of osteoclastic niches for tumor colonization in bone and, more importantly, reveals the potential of IL-20RB targeting with the neutralizing antibody in metastasis therapeutics.

## Methods

### Constructs and reagents.

Human *IL20RB* and murine *Il20rb* were constructed into pLVX-puro (Clontech) and pMSCV-neo (Clontech) vectors, respectively, for overexpression. For *IL20RB* and *CSF2* knockdown, the annealed sense and antisense shRNA oligonucleotides were cloned into the pLKO.1-puro vector (Addgene) with the following target sequences: CAGTGTACTATTCTGTCGAAT (shIL-20RB#2), CTCTGTACTCTCAACCAACAT (shIL-20RB#4), GGAGCTGCTCTCTCATGAAA (shCSF2#1), and CCCAGATTATCACCTTTGAAA (shCSF2#3). For *STAT3* knockdown, the annealed sense and antisense shRNA oligonucleotides were cloned into the pLKO.1-blasticidin vector (Addgene) with the following target sequences: CTCAGAGGATCCCGGAAATTT (shSTAT3#1) and GCACAATCTACGAAGAATCAA (shSTAT3#4). The antibodies used for Western blotting, immunoprecipitation, and immunohistochemistry were as follows: β-actin (catalog A2228, MilliporeSigma), GAPDH (catalog G9545, MilliporeSigma), IL-20RB (catalog PA549927, Thermo Fisher), IL-19 (catalog ab154187, Abcam, for Western blot), IL-19 (catalog PA546903, Thermo Fisher, for neutralizing), GM-CSF (catalog sc-32753, Santa Cruz Biotechnology Inc.), STAT3 (catalog 4904S, CST), phosphorylated STAT3 (p-STAT3) (catalog 9145S, CST), JAK1 (catalog 3344S, CST), p-JAK1 (catalog 3331S, CST), and GFP (catalog Abcam, ab13970). The human IL-19 recombinant protein was from Peprotech (catalog 200-19-10). The STAT3 inhibitors napabucasin (HY-13919) and stattic (HY-13818) were obtained from MedChemExpress. The ELISA kits used were as follows: GM-CSF (Proteintech; KE00003 human, KE10015 mouse), IL-19 (Abcam; ab231922 human, ab253220 mouse), IL-20 (Abcam, ab231927 human, ab235645 mouse), IL-24 (Abclonal, RK00112 human, RK04119 mouse). A549, H460, LLC HeLa, and 293T cells were purchased from the cell bank of Type Culture Collection of the Chinese Academy of Sciences.

### Osteoclastogenesis assays.

Osteoclastogenesis was conducted with bone marrow harvested from 4- to 7-week-old BALB/c mice. CM from cancer cells was mixed with α-MEM (supplied with 20% FBS, 25 ng/mL RANKL) at a 1:3 ratio for osteoclastic differentiation. After 5 to 7 days of culture, TRAP staining was performed. For CM collection, the primary mouse bone marrow cells were cultured with α-MEM (supplied with 20% FBS, 50 ng/mL RANKL). After 5 to 7 days of culture, the mature osteoclasts were washed by PBS and then cultured with RANKL-free α-MEM (supplied with 1% FBS) for 24 hours, after which the filtered supernatant was collected.

### TRAP staining.

TRAP staining was performed with the TRAP Kit (Sigma-Aldrich, 387A). Osteoclast numbers were assessed as multinucleated TRAP-positive cells per unit length.

### Luciferase dual-reporter assay.

A549 cells cultured in 48-well plates were transfected with the STAT3 firefly luciferase reporter plasmid, a Renilla luciferase plasmid, and the overexpression vector or control vector. Forty-eight hours later, the medium was discarded and cells were lysed with 60 μL luciferase lysis buffer (2 mM EDTA, 20 mM DTT, 10% glycerol, 1% Triton X-100 and 25 mM Tris-base, pH 7.8) for 1 hour at room temperature; 10 μL lysate was added with 30 μL firefly luciferase assay buffer (25 mM glycylglycine, 15 mM potassium phosphate, 15 mM MgSO4, 4 mM EGTA, 2 mM ATP, 10 mM DTT, and 1 mM d-luciferin, pH 7.8) or 30 μL Renilla luciferase assay buffer (0.5 M NaCl, 1 mM EDTA, 0.1 M potassium phosphate, 0.04% BSA, and 2 μM coelenterazine, pH 7.4). Then the luminescence was detected immediately by a Multimode Plate Reader (PerkinElmer).

### Organoid culture.

Cells were plated in Matrigel (BD Biosciences) and covered with organoid culture medium. A total of 500 to 1,000 cells were plated in each well of a 24-well plate; on days 7 to 14, the numbers of the organoids were analyzed. The components for lung cancer organoid culture medium are provided in Supplemental Table 1.

### In vitro EdU labeling.

Cells were cultured with 10 μM EdU for 24 hours and were stained with the Click Plus EdU 647 Imaging Kit (Thermo Fisher, C10640). The ratio of EdU^+^ cells was calculated.

### Neutralizing antibody production and purification.

IL-20RB–neutralizing monoantibody was developed by Abclonal Technology. Briefly, mice were immunized with the extracellular domain (amino acids 35–236) of human IL-20RB (XP_011511212). Splenocytes of immunized mice were separated and fused with SP20 tumor cells. Hybridoma were cultured with HybGro Medium (H630KJ, BasalMedia) supplied with CellTurbo supplement (H460JV, BasalMedia). For neutralizing antibody production, BALB/c mice were injected with 300 μL pristane (P9622, MilliporeSigma), and 2 × 10^6^ hybridoma cells were injected intraperitoneally 7 days after pristane injection. After 7 to 14 days, mice were sacrificed and ascites were collected. Antibody purification was performed with protein G resin (C600991, Sangon Biotech). Purified antibody was dialyzed into PBS and concentrated to 1 mg/mL.

### Co-IP analyses.

Cell lysates were centrifuged at 10,000 *g* for 15 minutes at 4°C to remove intact cells. The supernatant was either incubated with control IgG or primary antibody overnight in IP buffer (150 mM NaCl, 20 mM HEPES at pH 7.4, 1 % Triton X-100, 12.5 mM β-glycerophosphate, 1.5 mM MgCl_2_, 2 mM EGTA with phosphatase and protease inhibitors), followed by incubation with 20 μL of resuspended volume of protein A/G beads (GE Life Sciences) for 2 hours at 4°C to pull down bound proteins. Beads were centrifuged at 1,000 *g* for 5 minutes at 4°C to remove the supernatant, washed 4 times with the IP buffer, and boiled for 20 minutes at 95°C. Samples were run on SDS-PAGE gel, followed by Western blotting.

### Mouse experiments.

The C57BL/6 *Il19-*knockout mice were generated by Shanghai Model Organisms Center Inc. The BALB/c nude mice were purchased from Shanghai SLAC Laboratory Animal Co. for xenograft experiments. For bone metastasis analysis, 6-week-old male mice were used and 2 × 10^5^ cells were injected into the left ventricle of the heart. BLI data were acquired with an IVIS Spectrum CT system (PerkinElmer). Micro-CT data were acquired with a vivaCT80 (Scanco) system. For EdU-labeling assays, each mouse was intraperitoneally injected with 100 μg EdU (Thermo Fisher, C10640) 24 hours before bone harvest. The Click Plus EdU 647 Imaging Kit was used for EdU staining. The tumor cells were stained with a GFP antibody, and the ratio of EdU-positive cells to GFP-positive cells was calculated. IL-20RB–neutralizing antibody treatment was performed by intraperitoneal injection 1 week after tumor inoculation. Each mouse was injected with 100 μg IL-20RB–neutralizing antibody (LTMA1G11) or IgG control every other day. For blood component analysis, blood samples (50 μL) were collected using tubes, immediately diluted by PBS containing 5 mM EDTA into 100 μL, and analyzed by an Auto Hematology Analyzer (Mindray, BC-2800 Vet). No statistical method was used to predetermine the sample size of animal studies. Mice were randomly grouped, with approximately equal body weight between groups. No mice were excluded from analyses except those with unexpected death from nontumor reasons. Investigators were not blinded to allocation during the experiments and outcome assessment.

### Clinical analysis.

Samples from the SHTCH human lung cancer cohort, consisting of fresh-frozen (*n* = 99) and paraffin-embedded (*n* = 35) lung cancer tissues, were obtained from Shanghai Tenth People’s Hospital and Shanghai Chest Hospital. Basic demographic information for the cohort is shown in Supplemental Table 2. Paired para-carcinoma normal tissues of the same patients were also available as 20 fresh-frozen tissues. Bone metastatic lesions of lung cancer (*n* = 16) as well as paired primary lung tumor tissues and bone metastases of the same patients (*n* = 8 pairs) were also available in paraffin-embedded tissues. Bone metastasis of the patients was diagnosed by radiographic examination. RNA was extracted from the frozen tissues for *IL20RB* expression analysis. IL-20RB, IL-19, and p-STAT3 were immune stained, and the staining intensities were quantified using ImageJ (NIH).

### Data availability.

Sequence data of A549 and 5 derivative cell lines were deposited in the National Omics Data Encyclopedia (NODE OEP003002). Full, uncut gel images are available in the supplemental material.

### Statistics.

Data analyses were performed using GraphPad Prism 8.0 (GraphPad Software). The data presentation and statistical analyses are described in the figure legends. *P* values of less than 0.05 were considered statistically significant. *P* values were not corrected for multiple preplanned comparisons in experiments with more than 2 groups. The experiments in vitro were repeated independently multiple times with similar results, as indicated in the figure legends.

### Study approval.

All animal studies were conducted according to the NIH *Guide for the Care and Use of Laboratory Animals* (National Academies Press, 2011) and were approved by the Institutional Animal Care and Use Committee of Shanghai Institute of Nutrition and Health. Informed consent from all participants and approval from the Hospital’s Research Ethics Committee were obtained for retrieval of human tumor tissues from Shanghai Tenth People’s Hospital and Shanghai Chest Hospital.

## Author contributions

GH supervised this work. YH and GH drafted the manuscript. YH, WL, Y Liu, YW, CM, QW, PT, DH, ZJ, XL, XZ, YX, PZ, and Y Liang performed the experiments. HY, KX, FY, YSM, and DF contributed to clinical sample collection and analysis. DF and FY also helped the design of the project. All authors discussed the results and commented on the manuscript.

## Supplementary Material

Supplemental data

Supplemental table 1

## Version 1 08/25/2022

In-Press Preview

## Version 2 10/17/2022

Electronic publication
