# Supplementary material for: IL-20RB mediates tumoral response to osteoclastic niches and promotes bone metastasis of lung cancer
Source: J Clin Invest. 2022 Oct 17;132(20):e157917. doi: 10.1172/JCI157917 (PMC9566910; doi:10.1172/JCI157917)
Supplement: Supplemental data [file jci-132-157917-s036.pdf]

Supplemental Figures

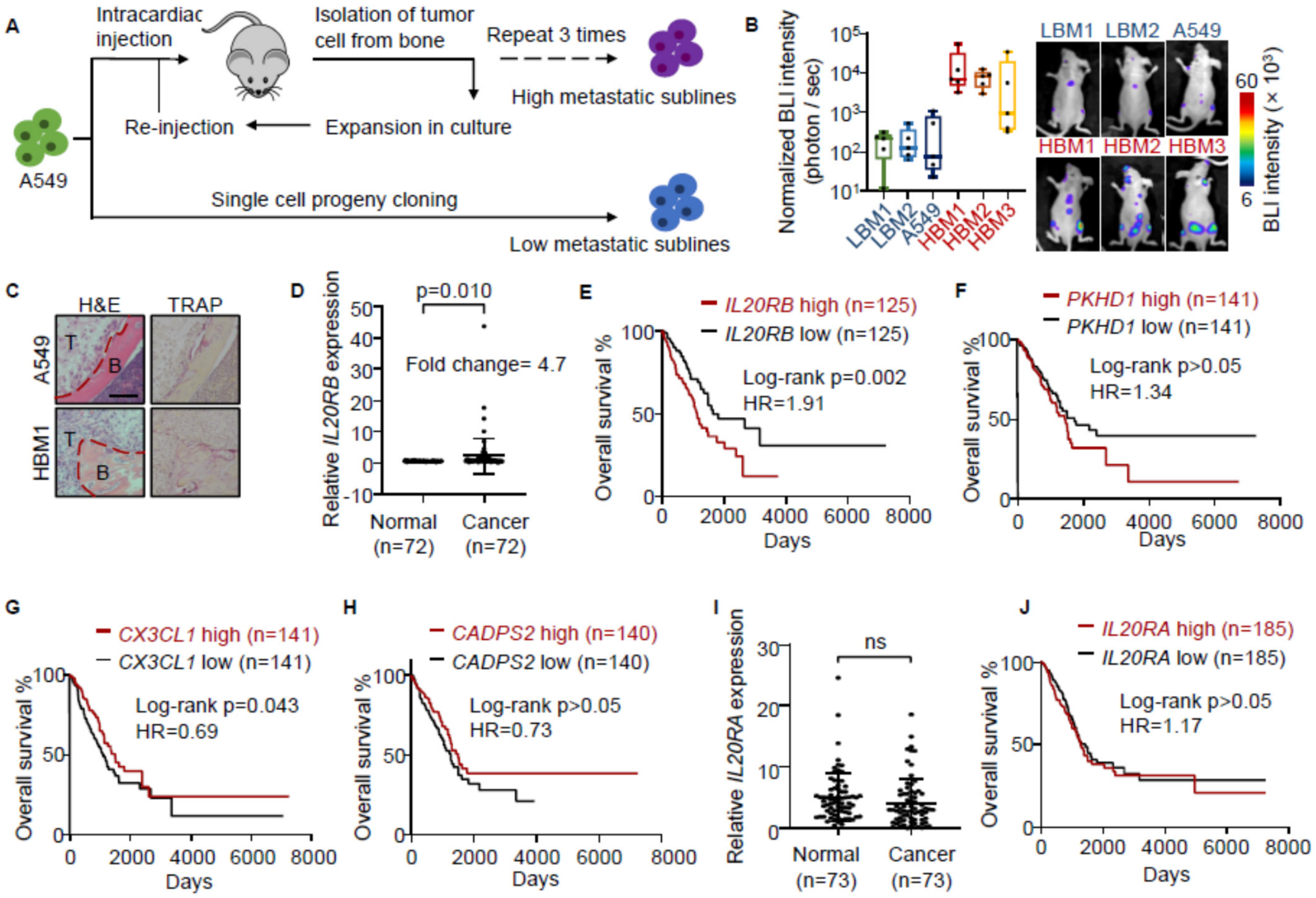

Supplemental Figure 1. Differentially expressed genes in A549 derivative cell lines with varied metastasis abilities to bone.

(**A**) Schematic of the subline derivation process.

(**B, C**) Intracardiac injection of A549 and other 5 derivative sublines for bone metastasis analysis. Shown are bioluminescent imaging (BLI) analysis of whole-body tumor burden of the mice (**B**, n = 5 mice per group), hematoxylin and eosin (H&E) and tartrate-resistant acid phosphatase (TRAP) staining of bone sections (**C**; T, tumor; B, bone, arrows point to osteoclasts).

(**D**) *IL20RB* expression in paired normal tissues and lung cancer tissues of the Korea lung cancer clinical dataset (1).

(**E-H**) Overall survival analyses of patients according to expression of *IL20RB* (**E**), *PKHD1* (**F**), *CX3CL1* (**G**) *and CADPS* (**H**) expression in the UCSC Xena lung cancer dataset (2).

(**I**) *IL20RA* expression in paired normal tissues and lung cancer tissues of the Korea dataset (1).

(**J**) Overall survival analyses of patients according to *IL20RA* expression in the UCSC Xena dataset (2).

Scale bar, 100 μm. *P* values were obtained by Mann-Whitney *U*-test (**B**), two-tailed paired *t*-test (**D, I**) and log-rank test (**E-H, J**); ns, not significant. Box plots display values of minimum, first quartile, median, third quartile, and maximum.

A

Score	Start	End	Strand	Sequence
16.2046	-2637	-2627	-	aagtaaacaga
12.36357	-2700	-2690	+	tggtaaacaac
10.62054	-1302	-1292	-	gagaaaacagc
9.350799	-698	-688	-	gaataaacatg
8.398483	-2341	-2331	-	atggaaacagg
8.112844	-1874	-1864	-	caataaacatt
8.069747	-1867	-1857	-	aagtatacaat
7.987784	-778	-768	+	gaataaataaa
7.929799	-553	-543	+	cagtaatcaaa
7.758451	-1071	-1061	+	ctaaaaacaac
7.123072	-1685	-1675	-	atataaataat
6.930044	-1893	-1883	+	tggtaaaaaga
6.849678	-462	-452	+	aggtaaccaat
6.740798	-1731	-1721	+	aaagaaacaca
6.715613	-451	-441	-	aaatgaacaag
6.141107	-941	-931	-	aaggaaacata
6.091776	-381	-371	-	gggtaaagaat
5.512441	-1434	-1424	-	agataaagaag
4.932144	-1885	-1875	+	agatatacagc
4.833182	-542	-532	+	cgatatacaaa
4.713123	-302	-292	+	tagaaaaaaga

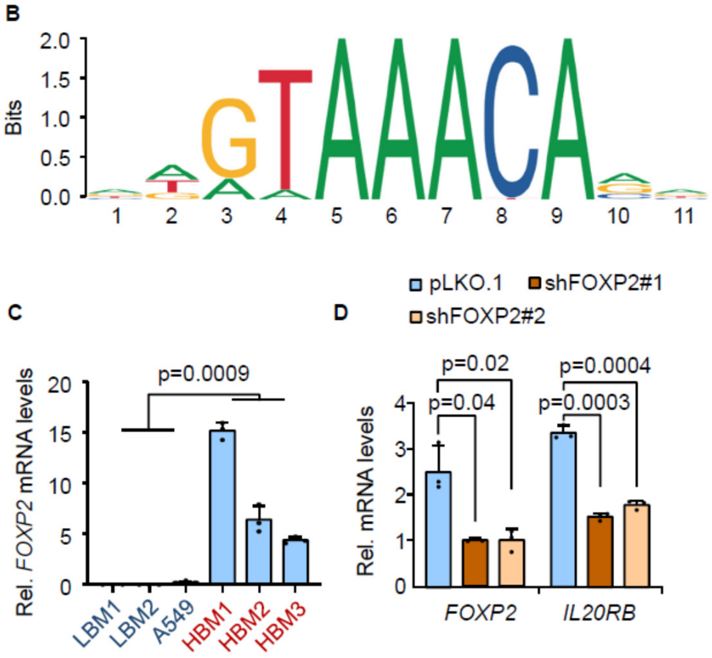

Supplemental Figure 2. *IL20RB* expression is regulated by FOXP2.

(**A, B**) Predicted binding sites of FOXP2 in the *IL20RB* promoter region in JASPAR database (**A**) and the FOXP2 binding motif (**B**).

(**C**) *FOXP2* mRNA levels in A549 derivative cell lines.

(**D**) *FOXP2* and *IL20RB* expression in HBM1 cells with *FOXP2* knockdown**.**

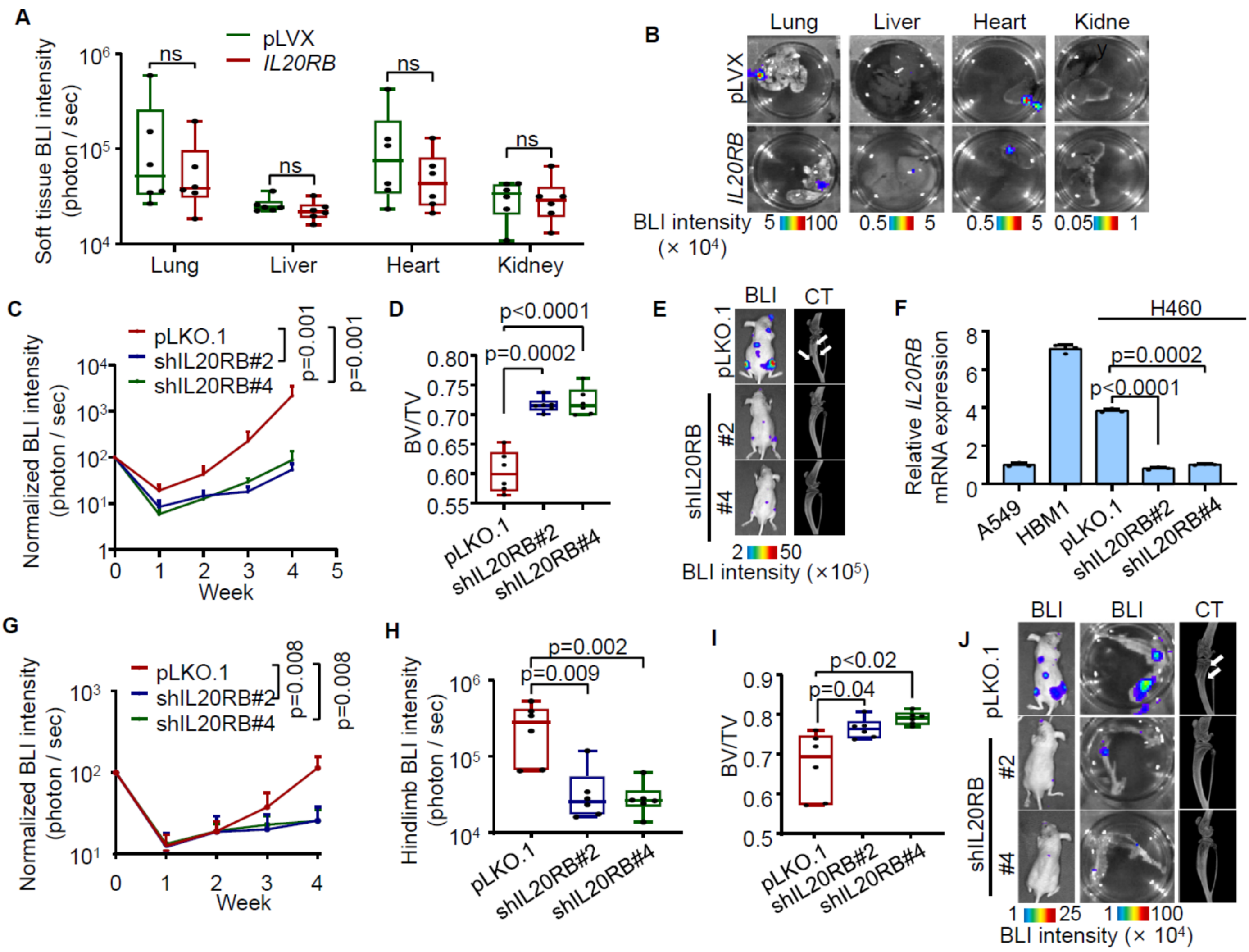

Supplemental Figure 3. IL20RB promotes bone metastasis of lung cancer. (**A, B**) Metastasis in various soft-tissue organs of mice with intracardiac injection of A549 with or without *IL20RB* overexpression. Shown are ex vivo BLI quantitation (**A**) and representative images (**B**) of different organs.

(**C-E**) Intracardiac injection of HBM1 with or without *IL20RB* knockdown for bone metastasis analysis (n = 6, 7, 7 mice for the 3 groups). Shown are weekly whole-body BLI quantification of tumor burden (**C**), micro-CT quantification of relative bone volumes of hindlimbs (**D**) and representative images of whole-body BLI and hindlimb micro-CT analyses (**E**, arrows point to osteolytic areas in the legs).

(**F**) *IL20RB* expression in A549 sublines and H460 with *IL20RB* knockdown.

(**G-J**) Intracardiac injection of H460 with or without *IL20RB* knockdown for bone metastasis analysis (n = 5 mice for each group). Shown are weekly whole-body BLI quantification of tumor burden (**G**), ex vivo BLI quantitation of hindlimbs (**H**),

micro-CT quantification of relative bone volumes of hindlimbs (**I**) and representative BLI and micro-CT images (**J**, arrows point to osteolytic areas in the legs).

P values were obtained by Mann-Whitney U-test (**A, C, G, H**) and two-tailed unpaired t-test (**D, F, I**); ns, not significant. Box plots display values of minimum, first quartile, median, third quartile, and maximum. Bar graphs are shown as mean ± s.d.

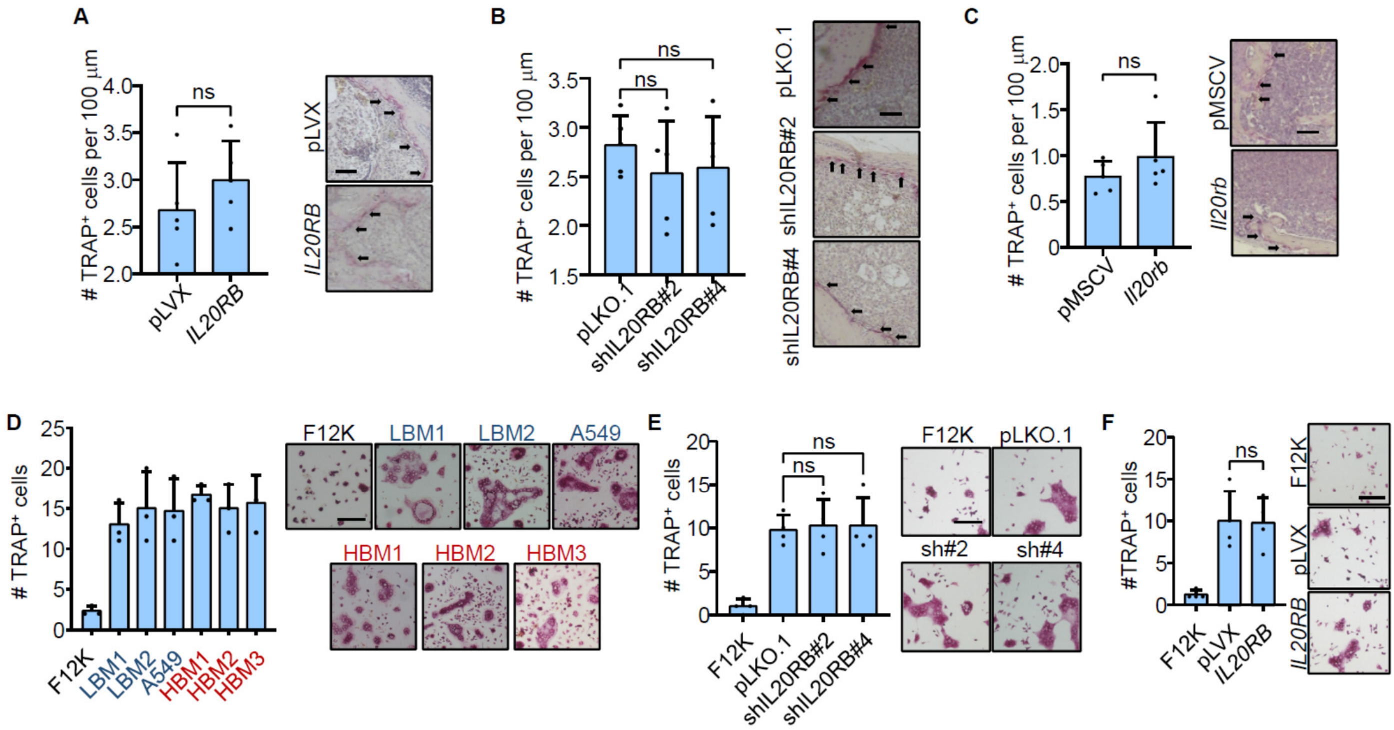

Supplemental Figure 4. IL20RB has no effects on osteoclastogenesis in vivo.

(**A-C**) Representative TRAP staining and quantification of osteoclasts along the tumor-bone interface of bone metastases caused by A549 cells with or without *IL20RB* overexpression (**A**), HBM1 cells with or without *IL20RB* knockdown (**B**), and LLC with or without *Il20rb* overexpression (**C**).

(**D-F**) Osteoclastogenesis assays from murine bone marrow when the bone marrow was treated with F12K empty medium or CM from various cancer cells, including A549 sublines (**D**), HBM1 with *IL20RB* knockdown (**E**) and A549 with *IL20RB* overexpression (**F**).

Scale bar, 100 µm. *P* values were obtained by two-tailed unpaired t-test; ns, not significant. Bar graphs are shown as mean ± s.d.

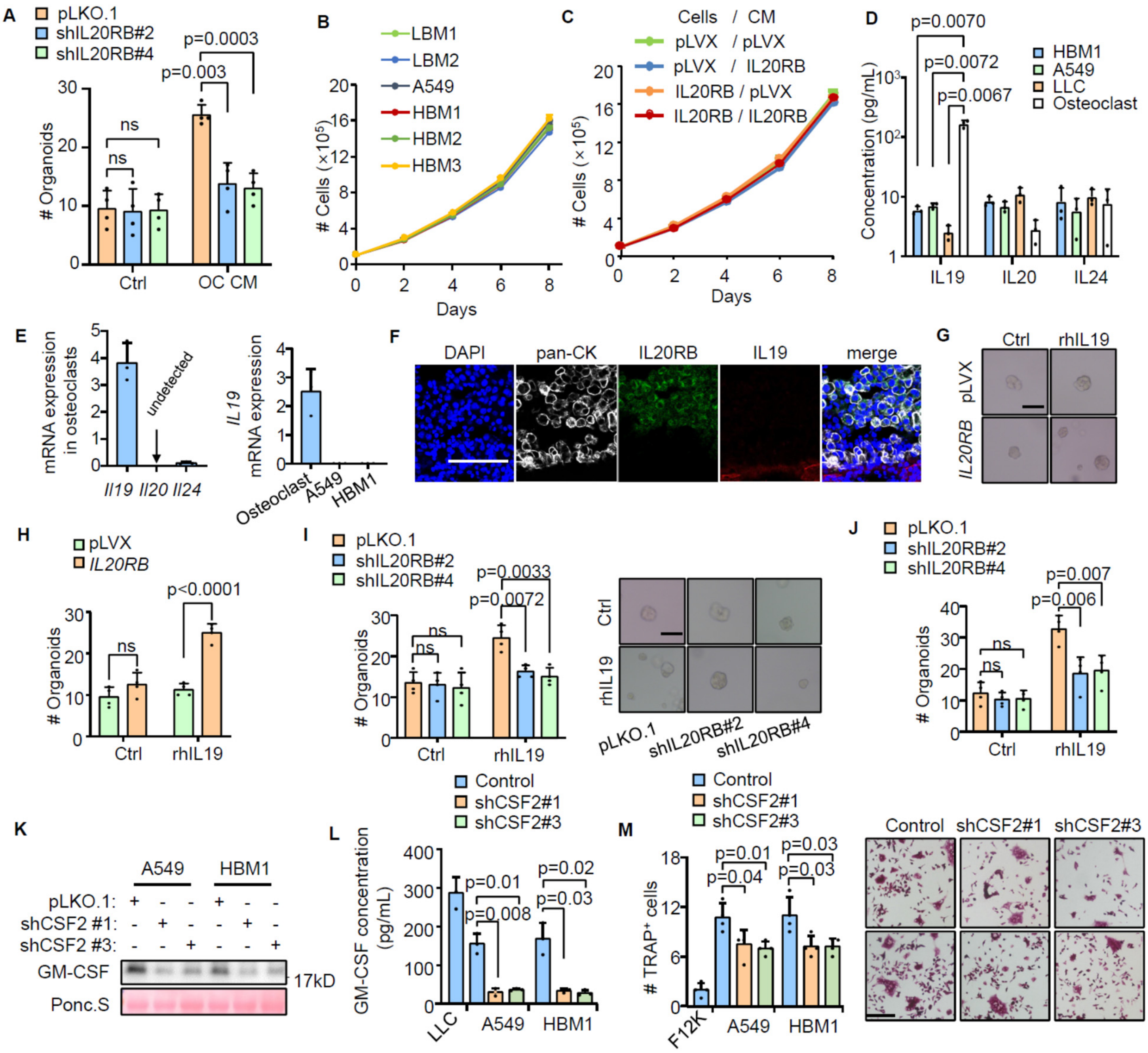

Supplemental Figure 5. The IL19-IL20RB axis mediates the responsiveness of tumor cells to osteoclasts.

(**A**) Organoid formation of H460 cells with IL20RB knockdown and/or treatment of OC CM.

(**B**) Growth curve of A549 sublines cultured in normal medium.

(**C**) Growth curve of control and *IL20RB*-overexpressing A549 cells cultured in their CM. CM was mixed with normal culture medium at a 1:3 ratio.

(**D**) ELISA analyses of IL19, IL20 and IL24 secreted by tumor cells and osteoclasts.

(**E**) Expression of IL20 subfamily cytokines in murine bone marrow-derived osteoclasts or tumor cells.

(**F**) IL19 and IL20RB immunostaining of bone metastases from lung cancer patients. Tumor cells were stained by pan-CK.

(**G, H**) Organoid formation of A549 with *IL20RB* overexpression and/or treatment of recombinant IL19 protein (**G**) and the representative organoid images (**H**).

(**I, J**) Organoid formation of HBM1 (**I**) or H460 (**J**) with *IL20RB* knockdown and/or treatment of recombinant IL19.

(**K**) GM-CSF secretion in A549 and HBM1 cells after *CSF2* knockdown.

(**L**) ELISA analysis of GM-CSF secretion in LLC, A549 and HBM1 with or without *CSF2* knockdown.

(**M**) Osteoclastogenesis assays from murine bone marrow when the bone marrow was treated with F12K empty medium or CM of A549 or HBM2 with *CSF2* knockdown.

Scale bar, 100 μm. *P* values were obtained by two-tailed unpaired t-test; ns, not significant. Bar graphs are shown as mean ± s.d.

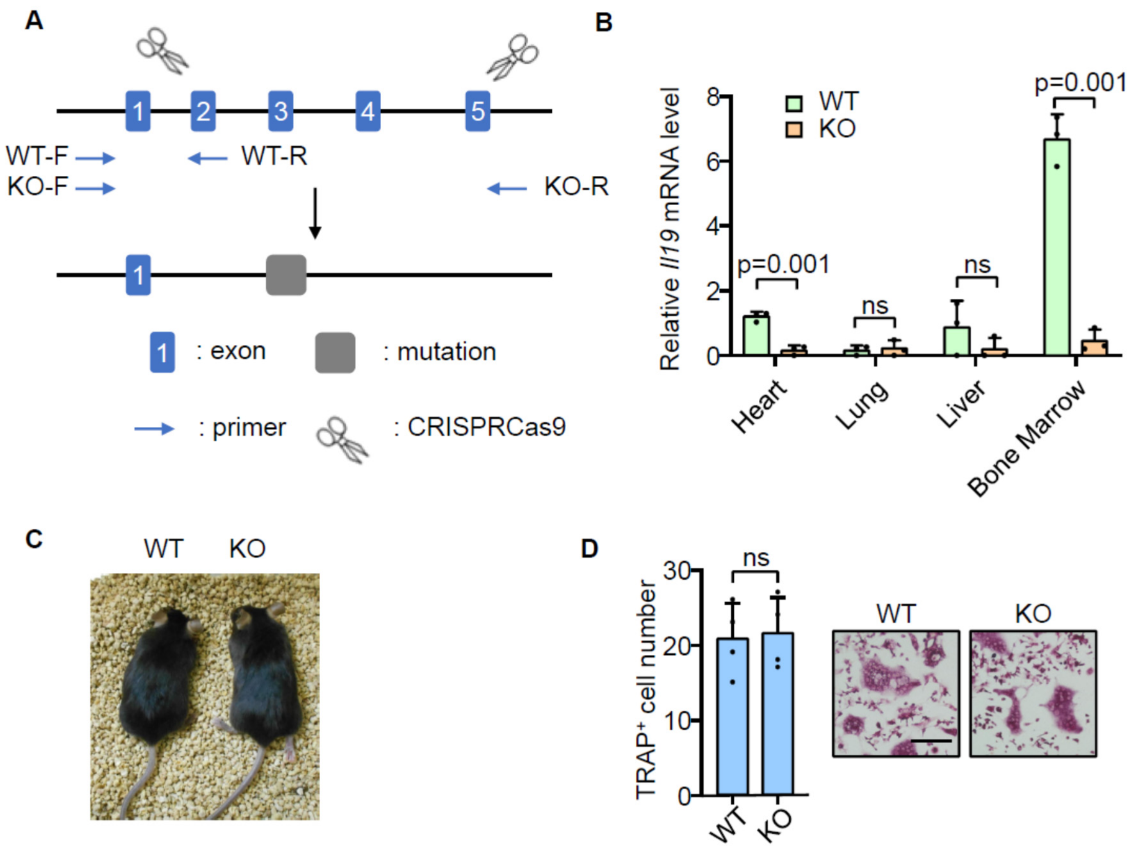

Supplemental Figure 6. The construction strategy and analysis of *Il19* knockout mice.

(**A**) Schematic of *Il19* knockout strategy.

(**B**) *Il19* mRNA expression in various tissues of wild type (WT) and *Il19* knockout (KO) mice (n = 3 mice per group).

(**C**) Representative images of WT/KO mice.

(**D**) Osteoclastogenesis of primary bone marrow cells of WT/KO mice.

Scale bar, 100 μm. *P* values were obtained by two-tailed unpaired t-test. Bar graphs are shown as mean ± s.d.

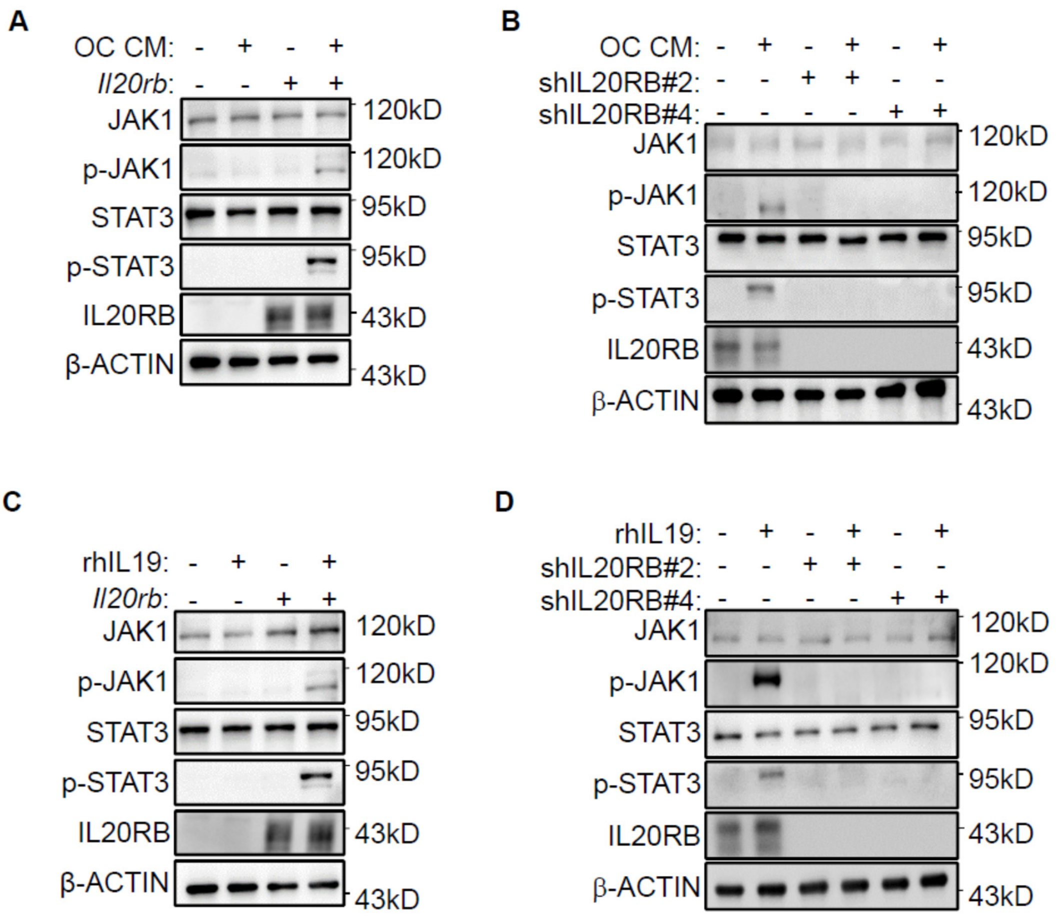

Supplemental Figure 7. Osteoclast-secreted IL19 activates JAK1-STAT3 in LLC and H460 cells.

(**A**) Phosphorylation of JAK1 and STAT3 in LLC cells with *Il20rb* overexpression and/or treatment of OC CM for 24 hours. OC CM was mixed with LLC culture medium at a 1:3 ratio.

(**B**) Phosphorylation of JAK1 and STAT3 in H460 cells with *IL20RB* knockdown and/or treatment of OC CM for 24 hours. OC CM was mixed with H460 culture medium at a 1:3 ratio.

(**C**) Phosphorylation of JAK1 and STAT3 in LLC cells with *Il20rb* overexpression and/or treatment of IL19 recombinant protein (25 ng/mL) for 15 min.

(**D**) Phosphorylation of JAK1 and STAT3 in H460 cells with *IL20RB* knockdown and/or treatment of IL19 recombinant protein (25 ng/mL) for 15 min.

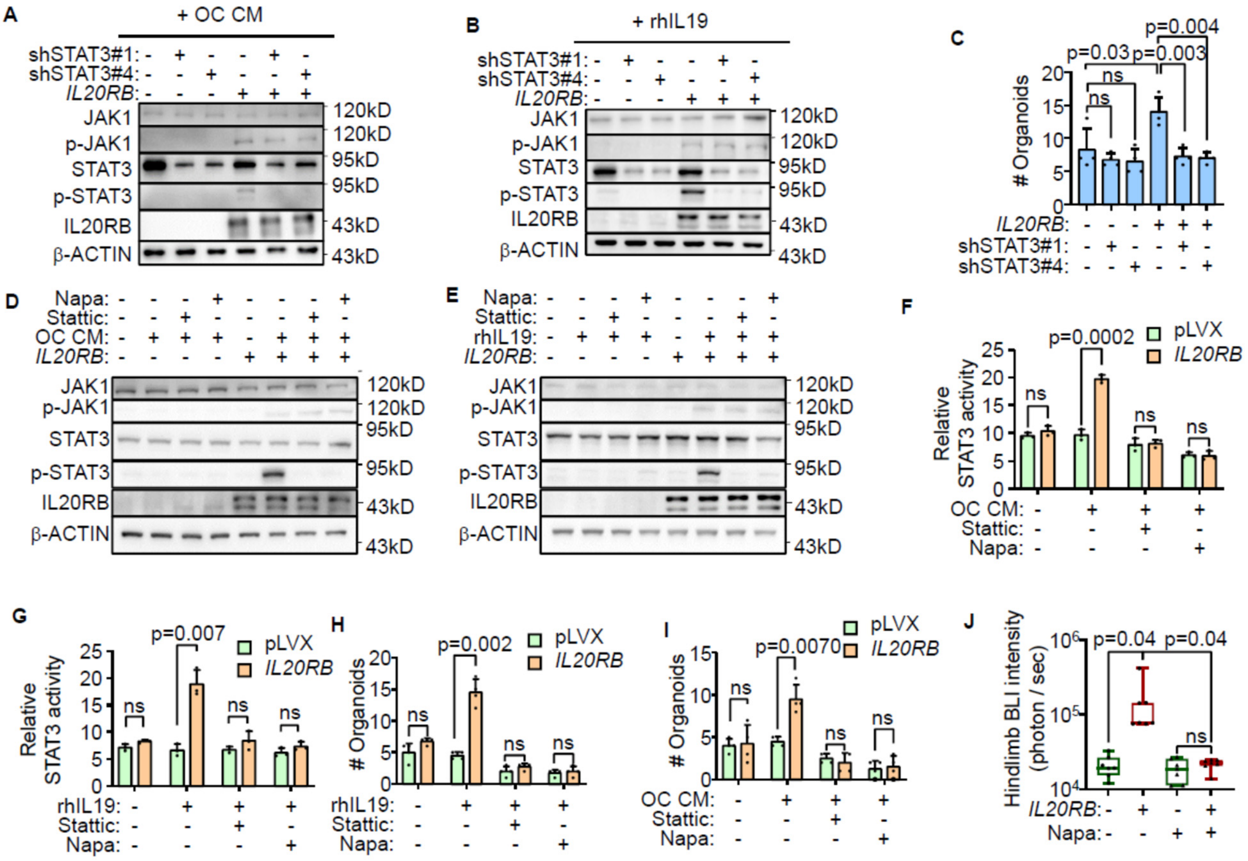

Supplemental Figure 8. STAT3 inhibitors suppress IL20RB-induced bone metastasis and tumor proliferation.

(**A, B**) Phosphorylation of JAK1 and STAT3 in A549 with *IL20RB* overexpression and/or *STAT3* knockdown after treatment with OC CM for 24 hours (**A**) or recombinant IL19 protein (25 ng/mL) for 15 min (**B**).

(**C**) Organoid formation of A549 cells with *IL20RB* overexpression and/or *STAT3* knockdown after treatment with recombinant IL19 protein (25 ng/mL).

(**D**) Phosphorylation of JAK1 and STAT3 in A549 cells with *IL20RB* overexpression and/or *STAT3* knockdown after treatment with Stattic (20 μM), Napabucasin (5 μM) and/or OC CM for 24 hours.

(**E**) Phosphorylation of JAK1 and STAT3 in A549 cells with *IL20RB* overexpression and/or *STAT3* knockdown after treatment with Stattic (20 μM), Napabucasin (5μM) and/or recombinant IL19 protein (25 ng/mL) for 15 min.

(**F**) STAT3-responsive reporter activity in A549 cells with *IL20RB* overexpression and/or *STAT3* knockdown after treatment with Stattic (20 μM), Napabucasin (5 μM) and/or OC CM for 24 hours.

(**G**) STAT3-responsive reporter activity in A549 cells with *IL20RB* overexpression and/or *STAT3* knockdown after treatment with Stattic (20 μM),

Napabucasin (5 μM) and/or recombinant IL19 protein (25 ng/mL) for 15 min.

(**H**) Organoid formation of A549 cells with *IL20RB* overexpression after treatment with Stattic (5 μM) or Napabucasin (0.05 μM) and recombinant IL19 protein (25 ng/mL).

(**I**) Organoid formation of A549 cells with *IL20RB* overexpression after treatment with Stattic (5 μM) or Napabucasin (0.05 μM) and OC CM.

(**J**) Ex vivo BLI quantification of tumor burden in hindlimbs of mice with inoculation of control and *IL20RB*-overexpression A549 cells and treatment of Napabucasin at week 4.

P values were obtained by Mann-Whitney *U*-test (**J**) and two-tailed unpaired t-test (**C, F-I**); ns, not significant. Box plots display values of minimum, first quartile, median, third quartile, and maximum. Bar graphs are shown as mean ± s.d.

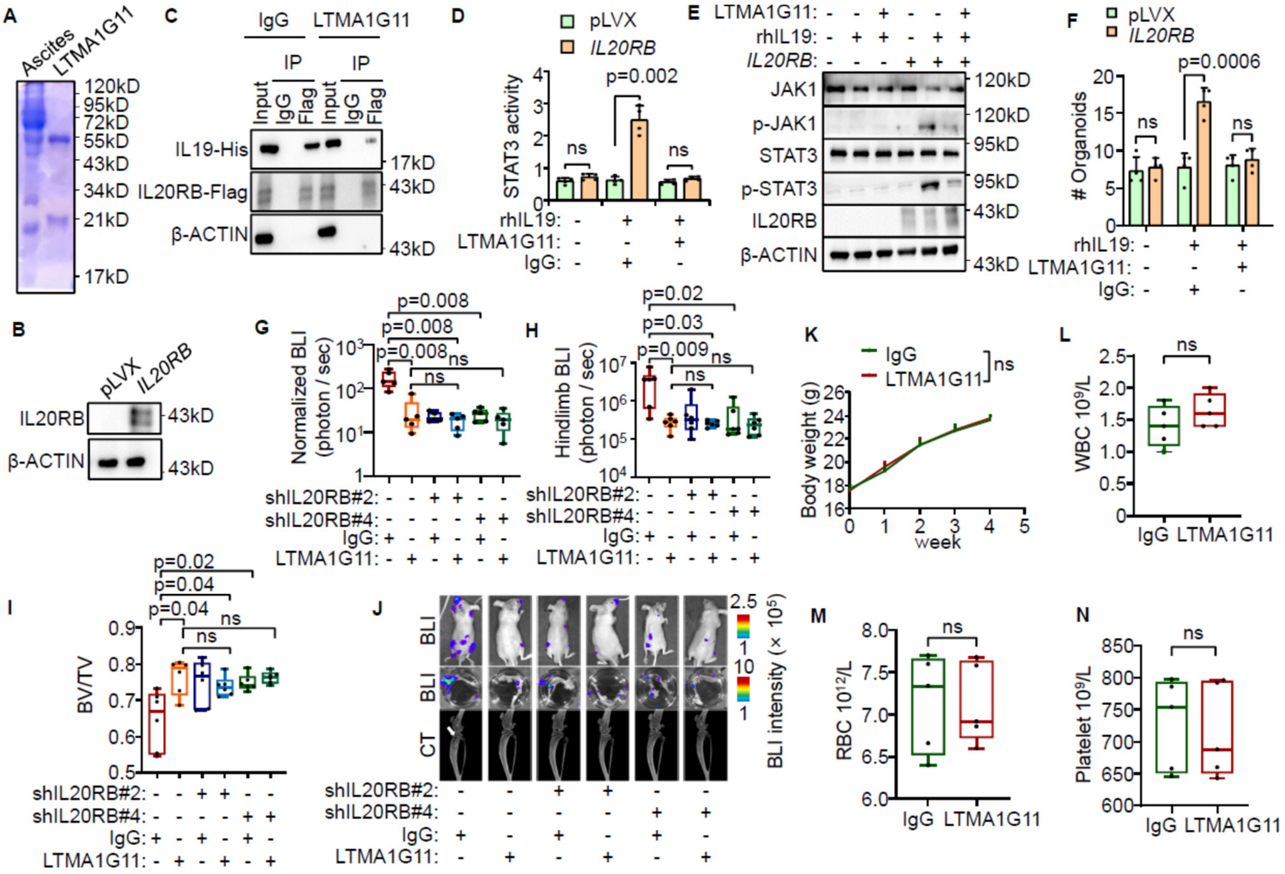

Supplemental Figure 9. Analysis of the IL20RB neutralizing antibody in vitro and in vivo.

(**A**) Coomassie blue staining of ascites from mice inoculated with the LTMA1G11 hybridoma and the purified antibody from the ascites.

(**B**) Western blotting of IL20RB in A549 with or without *IL20RB* overexpression by purified LTMA1G11.

(**C**) Co-IP of IL19 and IL20RB in the presence of LTMA1G11. His-tagged IL19 and Flag-tagged IL20RB were overexpressed in HeLa cells treated with LTMA1G11 (5 μg/mL) or control IgG (5 μg/mL). Cell lysates were immunoprecipitated with an anti-Flag antibody, followed by immunoblotting with anti-IL20RB and anti-IL19 antibodies.

(**D-F**) STAT3-responsive reporter activity (**D**), JAK1/STAT3 phosphorylation (**E**) and organoid formation (**F**) of A549 cells with or without *IL20RB* overexpression after treatment with LTMA1G11 (5 μg/mL) and/or recombinant IL19 protein (25 ng/mL) for 15 min.

(**G-J**) LTMA1G11 treatment of mice with intracardiac injection of HBM1 with or without *IL20RB* knockdown in mice for bone metastasis analysis. Shown are

BLI quantitation of the mice at week 4 after tumor inoculation (**G**, n = 5 mice per group), ex vivo hindlimb BLI quantitation (**H**), micro-CT quantification of relative bone volumes of hindlimbs (**I**), representative BLI and micro-CT images (**J**, arrows point to osteolytic areas in the legs)

(**K**) Body weights of healthy mice after continuous treatment with LTMA1G11 or control IgG.

(**L-N**) Blood components of healthy mice after continuous treatment with LTMA1G11 or control IgG. Shown are analyses of white blood cells (WBC, **L**), red blood cells (RBC, **M**) and platelets (**N**).

P values were obtained by Mann-Whitney *U*-test (**G, H**) and two-tailed unpaired t-test (**D, F, I, K-N**); ns, not significant. Box plots display values of minimum, first quartile, median, third quartile, and maximum. Bar graphs are shown as mean ± s.d.

References:

1. Derrien, T., *et al.* The GENCODE v7 catalog of human long noncoding RNAs: analysis of their gene structure, evolution, and expression. *Genome Res*, 2012; 22:1775-1789.

2. Goldman, M.J., *et al.* Visualizing and interpreting cancer genomics data via the Xena platform. *Nat Biotechnol*, 2020; 38:675-678.
